# Integration of microRNA changes *in vivo *identifies novel molecular features of muscle insulin resistance in type 2 diabetes

**DOI:** 10.1186/gm130

**Published:** 2010-02-01

**Authors:** Iain J Gallagher, Camilla Scheele, Pernille Keller, Anders R Nielsen, Judit Remenyi, Christian P Fischer, Karim Roder, John Babraj, Claes Wahlestedt, Gyorgy Hutvagner, Bente K Pedersen, James A Timmons

**Affiliations:** 1 Translational Biomedicine, Heriot-Watt University, Edinburgh, EH14 4AS, UK; 2 Centre for Inflammation and Metabolism, Department of Infectious Diseases and CMRC, Rigshospitalet, University of Copenhagen, DK2100, Denmark; 3 The Wenner-Gren Institute, Arrhenius Laboratories, Stockholm University, SE-106 91 Stockholm, Sweden; 4 Wellcome Trust Centre for Gene Regulation and Expression, College of Life Sciences, University of Dundee, Dundee, DD1 5EH, UK; 5 Department of Biochemistry, Scripps Research Institute, Jupiter, FL33458, USA; 6 Royal Veterinary College, University of London, Royal College Street, London, NW1, UK; 7 Centre for Healthy Ageing, Department of Biomedical Sciences, Panum Institutet, University of Copenhagen, Blegdamsvej 3B, DK-2200, Denmark

## Abstract

**Background:**

Skeletal muscle insulin resistance (IR) is considered a critical component of type II diabetes, yet to date IR has evaded characterization at the global gene expression level in humans. MicroRNAs (miRNAs) are considered fine-scale rheostats of protein-coding gene product abundance. The relative importance and mode of action of miRNAs in human complex diseases remains to be fully elucidated. We produce a global map of coding and non-coding RNAs in human muscle IR with the aim of identifying novel disease biomarkers.

**Methods:**

We profiled >47,000 mRNA sequences and >500 human miRNAs using gene-chips and 118 subjects (n = 71 patients versus n = 47 controls). A tissue-specific gene-ranking system was developed to stratify thousands of miRNA target-genes, removing false positives, yielding a weighted inhibitor score, which integrated the net impact of both up- and down-regulated miRNAs. Both informatic and protein detection validation was used to verify the predictions of *in vivo *changes.

**Results:**

The muscle mRNA transcriptome is invariant with respect to insulin or glucose homeostasis. In contrast, a third of miRNAs detected in muscle were altered in disease (n = 62), many changing prior to the onset of clinical diabetes. The novel ranking metric identified six canonical pathways with proven links to metabolic disease while the control data demonstrated no enrichment. The Benjamini-Hochberg adjusted Gene Ontology profile of the highest ranked targets was metabolic (*P *< 7.4 × 10^-8^), post-translational modification (*P *< 9.7 × 10^-5^) and developmental (*P *< 1.3 × 10^-6^) processes. Protein profiling of six development-related genes validated the predictions. Brain-derived neurotrophic factor protein was detectable only in muscle satellite cells and was increased in diabetes patients compared with controls, consistent with the observation that global miRNA changes were opposite from those found during myogenic differentiation.

**Conclusions:**

We provide evidence that IR in humans may be related to coordinated changes in multiple microRNAs, which act to target relevant signaling pathways. It would appear that miRNAs can produce marked changes in target protein abundance *in vivo *by working in a combinatorial manner. Thus, miRNA detection represents a new molecular biomarker strategy for insulin resistance, where micrograms of patient material is needed to monitor efficacy during drug or life-style interventions.

## Background

Skeletal muscle insulin resistance is an early feature during the progression towards type 2 diabetes (T2D) and is, in its own right, considered a risk factor for cardiovascular disease. While the defects in insulin-mediated glucose flux have been widely described, the global molecular characteristics of insulin resistant skeletal muscle have not. Four small gene-chip studies, relying on partial coverage of the human transcriptome, have attempted to define the global molecular basis of insulin resistance in human skeletal muscle [1-4]. While pioneering, neither the Yang *et al. *[4] nor Sreekumar *et al. *[3] studies were genome-wide, both studies suffered from small study populations, and the authors reported high false-positive rates. In the third and fourth studies, by Mootha *et al. *[1] and Patti *et al. *[2], a coordinated down-regulation of oxidative phosphorylation related (OXPHOS) genes in the skeletal muscle of patients was the only change reported and this was proposed to be the underlying cause of skeletal muscle insulin resistance [5-7]. Indeed, 'subset' analysis of a collection of genes (for example, 200 to 400) has become a powerful approach to detecting coordinated defects in biological pathways *in vivo*, and this method has made important contributions to the systems biology field. A separate line of investigation by Petersen *et al. *[8,9] introduced a magnetic resonance spectroscopy approach [10] to study insulin resistance *in vivo *[11]. This method estimates unidirectional ATP synthesis, but it is unclear if it has been validated to take into account the multiple assumptions that allow net ATP generation to be calculated [12,13]. Despite the clear caveats and continuing debate in the field [14,15], the concept of an OXPHOS impairment [5,16,17] is widely accepted. Nevertheless, a clear explanation for the general lack of mRNA abundance changes, beyond *OXPHOS *mRNAs, still remains to be explained. One thing that it is certainly not due to is the lack of sensitivity of gene-chip technology as it readily detects high and low abundance RNA molecules under a variety of conditions [18-20]. In addition, the general lack of a global transcriptional signature has been a consistent finding.

Non-coding RNA has emerged in recent years [21] as being of functional importance [22]. In particular, microRNAs (miRNAs) are accepted regulators of mammalian cell phenotype [23-25]. miRNAs are approximately 22-nucleotide post-transcriptional regulators of gene product abundance, able to block the translation of protein-coding genes [26]. miRNAs regulate development and differentiation [27,28] and brain and skeletal muscle tissue have the most abundant expression of tissue-specific miRNA species [29]. miRNAs have been implicated in the regulation of metabolism [27,30] and insulin secretion [31] while expression is altered in extreme muscle disorders [20,32]. Whether miRNAs are altered during the development of diabetes or skeletal muscle insulin resistance in humans is unknown, and there are still very few studies characterizing miRNA changes *in vivo*, in humans. The molecular rules governing the targeting of each miRNA to individual genes have been documented [25,33] and help identify which protein coding genes are targeted when a single miRNA is modulated in a cell [23,24]. In contrast, multiple changes in miRNA abundance can occur *in vivo *[32], where simultaneously up-regulated and down-regulated miRNAs can target the same gene but with a range of predicted efficacies [25]. To date no study has established the net biological impact of multiple miRNA changes *in vivo*.

In the present study we devised a new strategy for predicting which proteins and biological pathways would be altered *in vivo *under such circumstances (Figure S1 in Additional file 1). Our approach was built on the *in vitro *molecular rules encompassed by the site-specific context score criteria, as these criteria can significantly enrich a gene list in genuine targets when a single miRNA is studied in a cell-based system [34]. Using three to nine times the number of human subjects (n = 118) as previous studies [1-4] and a more comprehensive 'genome-wide' RNA profiling strategy (>47,000 mRNA sequences, and >500 miRNA sequences), we aimed to identify the global molecular nature of skeletal muscle insulin resistance in human T2D and provide new bioinformatic and protein level validation for our conclusions.

## Methods

We recruited 118 subjects for the study (Table 1) and the degree of insulin resistance was verified by applying the World Health Organization diagnostic criteria for diabetes [35]. Exclusion criteria were treatment with insulin, recent or ongoing infection, history of malignant disease or treatment with anti-inflammatory drugs. The cohort consisted of approximately 65% male and 35% female subjects. Participants were given both oral and written information about the experimental procedures before giving their written, informed consent. The study was approved by the Ethical Committee of Copenhagen and Frederiksberg Communities, Denmark (j.nr (KF) 01-141/04), and performed according to the Declaration of Helsinki.

**Table 1:** Characteristics of the 3 subject populations in the study

	T2D (n = 45)	IGT (n = 26)	NGT (n = 47)
Age	54.8 ± 10.2	56.4 ± 10.7	51.3 ± 10.7
BMI	31.4 ± 6.2	30.9 ± 6.1	31.1 ± 7.2
VO_2max_	26.9 ± 8.4	28.2 ± 9.7	29.5 ± 10.5
Fasting glucose	9.8 ± 4.4*	5.9 ± 0.5^†^	5.0 ± 0.4
Fasting insulin	91.2 ± 8.9^†^	88.2 ± 13.5^‡^	56.6 ± 8.3
HOMA1_log_	0.67 ± 0.07*	0.46 ± 0.05*	0.20 ± 0.05
2-h glucose (OGTT)	17.9 ± 5.5*	7.4 ± 2.4^†^	5.5 ± 1.2
HbA1c	7.4 ± 1.8*	5.8 ± 0.3^†^	5.5 ± 0.2

Data are mean ± standard deviation. BMI, body mass index; VO_2max_, ml/kg/minute; Fasting glucose and 2-h glucose tolerance is mmol/L; HbA1c is percentage glycosylated hemoglobin. *P < 0.001 when compared with either NGT or IGT; ^†^*P *< 0.01 when compared with the NGT group; ^‡^*P *= 0.07 when compared with the NGT group. OGTT, oral glucose tolerance test.

### Clinical evaluation protocol

Participants reported between 8 and 10 am to the laboratory after an overnight fast. Subjects did not take their usual medication for 24 hours preceding the examination, and T2D subjects did not take hypoglycemic medicine for 1 week prior to examination. Note that the correlation between fasting glucose and hbA1c remained high (R^2 ^= 0.71; Additional file 2), indicating that short-term glucose homeostasis did not appear greatly disrupted by the 1-week drug withdrawal. Body mass and height were determined for body mass index (BMI) calculations. The subjects performed an oral glucose tolerance test and an aerobic capacity test. Peak aerobic capacity was determined by the Åstrand-Ryhming indirect test of maximal oxygen uptake (VO_2max_) [36].

### Blood analyses and oral glucose tolerance test

Blood samples were drawn before and 1 and 2 hours after drinking 500 ml of water containing 75 g of dissolved glucose. The World Health Organization diagnostic criteria were applied, as were calculations of insulin resistance (homeostatic model assessment (HOMA)). Plasma was obtained by drawing blood samples into glass tubes containing EDTA and serum was obtained by drawing blood into glass tubes containing a clot-inducing plug. The tubes were immediately spun at 3,500 g for 15 minutes at 4°C and the supernatant was isolated and stored at -20°C until analyses were performed. Plasma glucose was determined using an automatic analyzer (Cobas Fara, Roche, France). All samples and standards were run as duplicates and the mean of the duplicates was used in the statistical analyses.

### Muscle tissue biopsies

Muscle biopsies were obtained from the *vastus lateralis *using the percutaneous needle method with suction [37]. Prior to each biopsy, local anesthetic (lidocaine, 20 mg ml^-1^; SAD, Denmark) was applied to the skin and superficial fascia of the biopsy site. Visible blood contamination was carefully removed and all biopsies were frozen in liquid nitrogen and subsequently stored at -80°C until further analysis. RNA extraction was carried out using TRIzol (Invitrogen, Carlsbad, CA, USA) and a motor-driven homogenizer (Polytron, Kinematica, Newark, NJ, USA) as described [38].

### Affymetrix microarray

Hybridization, washing, staining and scanning of the arrays were performed according to manufacturer's instructions (Affymetrix, Inc. [39]). We utilized the Affymetrix U133+2 array platform and 15 μg of cRNA was loaded onto each chip. All array data were normalized using the Microarray Suite version 5.0 (MAS 5.0) algorithm to a global scaling intensity of 100. Arrays were examined using hierarchical clustering to identify outliers prior to statistical analysis, in addition to the standard quality assessments, including scaling factors and NUSE plot. No array included in this analysis failed these standard quality assurance procedures. We relied on several statistical approaches to analyze the data with and without pre-filtering of gene lists. We utilized custom chip definition files (CDFs) [40] to improve the annotation precision [41]. Using the MAS 5.0-generated present-absent calls improves the sensitivity of the differential gene expression analysis [42] as it increases the statistical power of the analysis. We chose to remove probe sets that were declared 'absent' across all chips in the study. The microarray data were subjected to global normalization using the robust multi-array average expression measure (RMA) in the Bioconductor suite [43] and analyses were compared in parallel with MAS 5.0-based normalization, following the negative result (see below) with the MAS 5.0 data. The CEL files have been deposited at the Gene Expression Omnibus under reference number [GEO:GSE18732] and patient phenotype data have also been made available at the same location and with this manuscript.

### miRNA microarrays

Total RNA was pooled from groups of subjects with similar clinical profiles from the larger cohort. This was done to generate sufficient RNA for labeling and the average clinical profile of the subjects that contributed to the miRNA analysis can be found in Table S1 in Additional file 1. Each sub-pool was >2 μg and 4 independent miRNA profiles per clinical subgroup were created (resulting in a total of 16 independent miRNA determinations per clinical condition). The microarrays were miRCURY™ v10.0 LNA miRNA array from Exiqon (Vedbaek, Denmark). The Exiqon probe set consists of 1,700 custom made capture probes that are enhanced using locked nucleic acid (LNA) technology, which is claimed to normalize the Tm of the capture probes, as insertion of one LNA molecule into the capture probes increases the Tm by 2 to 8°C. Total RNA (2 μg) was labeled with Hy3 dye according to the manufacturer's protocol using the labeling kit from Exiqon. For the labeling reaction, RNA was incubated with the Hy3 dye, labeling enzyme and spike-in miRNAs, in a total volume of 12.5 μl, for 1 hour at 16°C. The enzyme was then heat-inactivated at 65°C for 15 minutes. The samples were incubated at 95°C for 2 minutes, protected from light. A total of 32.5 μl of hybridization buffer was added to make up the volume required by the hybridization station. The samples were briefly spun down and filtered through a 0.45-micron durapore filter (Millipore, Billerica, USA). Samples were then loaded onto the MAUI (BioMicro Inc., Salt Lake City, UT, USA) hybridization station. The arrays were incubated at 56°C for 16 hours, then washed briefly in 60°C using buffer A, rinsed in buffer B, followed by a 2-minute wash in buffer B and a 2-minute wash in buffer C. The arrays were spun for 5 minutes at 1,000 rpm followed by immediate scanning using a GenePix 4200A microarray scanner. Data were analyzed using GenePix Pro 6^® ^software. Following quantile normalization of the entire chip, the distribution of intensities was plotted for all of the human annotated miRNA probes and this was compared with background signal intensities, with a cutoff of 400 units being taken as an expressed miRNA (total of 171 human miRNAs). Differential expression was determined using the significance of microarray analysis (SAM) approach and miRNAs with a false discovery rate (FDR) of 10% or better and modulated by >30% were selected for further validation studies. Quantile normalized raw data can be found in Additional file 2. Changes were verified using the Applied Biosystems TaqMan assays (Applied Biosystems, Foster City, CA, USA) on individual patient samples (Table S1 in Additional file 1; n = 10 for each patient group) and pooled RNA for Northern blots (where stated).

### Real time quantitative PCR detection of mature miRNAs in skeletal muscle

Individual muscle RNA samples from 30 subjects (Table S1 in Additional file 1) were used for detection of individual miRNA expression. Subjects were matched to have identical age, BMI and maximal oxygen uptake (VO_2max_); note that we profiled only non-obese subjects for resource reasons. The Taqman^® ^MicroRNA assay (Applied Biosystems), which detects mature miRNA, was used to measure miR-1 (Cat#4373161), miR-133a (Cat#4373142), miR-133b (Cat# 4373172) and miR-206 (Cat#4373092). The assay relies on a miRNA-specific looped primer for the reverse transcription (RT) reaction, which extends the mature miRNA sequence and enables detection in the subsequent Taqman assay. It is possible for the RT step to amplify the closely related pre-miRNA sequence. However, in competition with a more efficiently amplified, primer extended mature miRNA, an insignificant contribution from the pre-miRNA to the real time PCR signal is expected (approximately 1 to 5%) [44,45].

For each miRNA RT-PCR reaction, 5 ng of total RNA was reverse transcribed using the TaqMan^® ^MicroRNA Reverse Transcription Kit (Applied Biosystems, PN4366597) and miRNA-specific primers. For quantitative real-time PCR (qPCR) the TaqMan^® ^2X Universal PCR Master Mix No AmpErase^® ^UNG was used (Applied Biosystems, PN4324020). The samples were run on a 7900 Fast Real-Time PCR System (Applied Biosystems) on the 9600 emulation mode in triplicates of 10 μl per well. The miRNA expression levels were normalized to the small nuclear RNA RNU48 (Cat#4373383), which appears not to vary between subject samples for human skeletal muscle (using 18S as a comparator for RNU48). All reactions were run single-plex in triplicate and quantified using the ΔCt method. Data are analyzed using ANOVA to compare differences in ΔCt values between the three groups followed by a *post hoc t*-test where appropriate to identify specific group differences. For all analyses *P *< 0.05 was considered significant. Statistical calculations were performed using SPSS (SPSS Inc, Chicago, IL, USA) or Sigmastat (Systat Software Inc, San Jose, CA, USA).

### Detection of pri-miRNA expression using SYBR green qPCR

To determine if pri-miRNA transcript abundance differs across the presumed polycistronic mir-1/mir-133a pri-miRNA, we utilized qPCR. Reverse transcription was performed on 1 μg RNA in a reaction volume of 40 μl using the high capacity cDNA reverse transcription kit (Applied Biosystems) and random hexamers. The RT reaction was run at 25°C for 10 minutes, 37°C for 120 minutes, and 85°C for 5 s. SYBR green reagents (Applied Biosystems) were used for detection of the pri-miRNA transcripts. Primers were designed to amplify the genomic region near the pre-miRNA hairpin to determine whether 'neighboring' pri-miRNAs are expressed in a similar manner. Primer sequences are listed in Table S2 in Additional file 1. Primer efficiency was established by plotting a standard curve of Ct values from serial dilutions of cDNA and these were similar in all cases. Each qPCR reaction was prepared using 6 μl SYBR green mastermix, 4.6 μl nuclease-free H_2_O, 30 nM forward primer, 30 nM reverse primer and 1.2 μl of a 1:10 cDNA dilution in a total volume of 10 μl. The PCR reaction was run on an Applied Biosystems 7900 Fast Real-Time PCR system in standard mode, 10 minutes at 95°C, then 45 cycles consisting of 15 s at 95°C and 60 s at 60°C. Ct values for triplicates were averaged and ΔCt values computed using 18S as the control.

### Northern blot to detect pre- and mature miRNA

To enable detection by Northern blotting, RNA was pooled from each of the three groups above to provide independent pools of 10 μg of total RNA. An oligonucleotide was synthesized to probe for miR-133a/b (5'-AGCUGGUUGAAGGGGACCAAA-3'). A small RNA blot was prepared using a 15% denaturing gel, consisting of 15 ml SequaFlowGel sequencing system concentrate, 7.5 ml SequaFlowGel diluent, 2.5 ml 10× MOPS buffer, 250 μl 10% ammonium persulfate (Sigma, Poole, Dorset, UK) and 25 μl tetramethylethylenediamine. RNA was dissolved in 2× formamide loading dye, incubated at 95°C for 2 minutes and loaded onto the gel along with Decade Marker (AM7778, Applied Biosystems). The gel was pre-heated and then run at 100V for 3 hours using the WB system (Invitrogen) with 1× MOPS/NaOH (20 mM, pH 7.0) running buffer. The RNA was transferred to a HybondN neutral membrane (Amersham Biosciences, Little Chalforn, Bucks, UK) by applying a current of 400 mA for 1 to 1.5 hours. For chemical cross-linking [46] the membrane was incubated at 55°C for 2 hours in a cross-linking solution consisting of 9 ml RNase free water, 245 μl 1-methylimidazole, 300 μl 1 M HCl and 0.753 g EDC (N-Ethyl-N'-(3-dimethylaminopropyl)carbodiimide hydrochloride). After membrane incubation at 37°C for 1 hour in a pre-hybridization mix (12.5 ml formamide, 6.25 ml SSPE (20×), 1.25 ml Denhardt (100×), 1.25 ml 10% SDS and 500 μl herring sperm (hs)DNA (2 mg/ml)) hybridization occurred overnight in a solution of 1 μl 50 μM oligo, 11 μl nuclease-free water, 2 μl 10× buffer, 2 μl RNase inhibitor, 2 μl T4 PNK (polynucleotide kinase) and 2 μl ^32^P-j-ATP that had been incubated at 37°C for 1 hour and filtered through a G-25 column. The membrane was then washed twice in 2× SSC and 0.1% SDS for 1.5 hour at 65°C and hybridization was detected by Kodak photographic film. The membrane was subsequently stripped and re-probed for tRNA as a loading control.

### miRNA knockdown and western blot analysis in C2C12 myoblasts

C2C12 cells were seeded at 50% confluency in Dulbecco's modified Eagle's medium (DMEM) and 10% fetal calf serum (FCS). Before transfection cells were transferred to the serum and antibiotic free medium Optimem (Invitrogen), and transfected with 100 nM LNA miRNA inhibitors or scrambled oligo (Exiqon) with Oligofectamine (Invitrogen) following the manufacturer's protocol. Four hours after the transfection, FCS was added back to a final concentration of 8%. After 48 hours the cells were lysed, and RNA and protein were isolated and retained for further analysis. Cells were lysed by boiling in Laemmli buffer for 5 minutes. Insoluble material was removed by centrifugation and protein content quantified using the BCA reagent (Pierce, Little Chalforn, Bucks, UK). Proteins were size fractionated by SDS-PAGE using a 4 to 12% gradient bis-Tris NuPage gel (Invitrogen) and transferred onto a nitrocellulose membrane (Whatman, Little Chalforn, Bucks, UK). The efficacy of the transfer was examined by Ponceau Red staining of the membrane. The membrane was blocked by incubation at room temperature with a solution of 5% skimmed milk in Tris-buffered saline (TBS), 0.2% Tween, 0.05% Triton X100 (TBST) or 5% bovine serum albumin (BSA) in TBST. Incubation with primary antibody anti-PTBP1 (Polypyrimidine tract-binding protein 1; Proteintech Group Inc. (Chicago, Illinois, USA) at 1:1,000 in 5% skimmed milk/TBST or anti-CDC42 (Cell Signaling Technology, Danvers, MA, USA) at 1:1,000 in 5% BSA/TBST) took place overnight at 4°C. Blots were washed and incubated with an anti-rabbit IgG horse radish peroxidase-conjugated antibody (1:5,000; Cell Signaling Technology) for 1 hour at room temperature. Specific signal was detected using the ECL reagent (GE Healthcare, Little Chalforn, Bucks, UK) and exposure on Kodak BioLight film. An image of the Ponceau membrane and each blot were analyzed using the ImageJ software (NIH). The area under the curve for each blot signal was corrected for protein loading using the area under the curve from the Ponceau signal. These loading corrected signals were then scaled to the signal for the cells transfected with scrambled sequence and percentage changes in signal were calculated. A minimum of two independent cell transfections were carried out.

### Muscle tissue western blot analysis

Human muscle samples were homogenized (n = 13) using a Tissue-lyser (Qiagen, Crawley West Sussex, UK) in 50 mM Tris-HCl, pH 7.4, 150 mM NaCl, 1 mM EGTA, 1 mM EDTA, 0.25% NaDeoxycholate, 1% Triton X-100. Phosphatase inhibitor cocktail 1 and 2 (Sigma Aldrich, Poole, Dorset, UK) and protease inhibitor complete mini (Roche, Welwyn Garden City

Hertfordshire, UK) was added to the buffer immediately before homogenization. Following homogenization, protein lysates were centrifuged at maximum speed for 1 hour at 4°C and the pellet was discarded. Protein concentration was measured using a Bio-Rad protein assay. Samples were diluted in 5× Laemmli buffer and boiled for 2 minutes before subsequent loading of 25 μg onto a 4 to 12% gradient bis-Tris NuPage gel (Invitrogen). The gel was run for 120 minutes at 125V and protein was transferred onto a PVDF membrane using a semi-dry blotting system for 2 hours at 20V (Invitrogen). The membrane was blocked for 1 hour at room temperature in 5% skimmed milk. Incubation with primary antibody took place overnight at 4°C. Antibody dilutions were: anti-PTBP1 at 1:4,000 in 5% skimmed milk/TBST; anti-CDC42 at 1:4,000 in 5% BSA/TBST; anti-HOXA3 (Abnova, Walnut, CA, USA) at 1:2,000 in 5% milk; anti-HOXC8 (Abnova) 1:1,000 in 5% milk; anti-BIM at 1:2,000 in 5% BSA; and anti-BDNF (Brain-derived neurotrophic factor; Santa Cruz, Santa Cruz, CA, USA) at 1:200 in 0.25% BSA. Blots were washed and incubated with anti-rabbit or anti-mouse IgG horse radish peroxidase-conjugated antibody (1:2,000; Cell Signaling Technology) for 1 hour at room temperature. The signal was detected using Supersignal West Femto Luminal/Enhancer Solution (Thermo Scientific, Waltham, MA, USA) and subsequent exposure in a charge-coupled device camera (Bio-Rad, Hemel Hempstead

Hertfordshire, UK). Following exposure, blots were briefly rinsed in TBST and then incubated in 0.5% Reactive Brown (Sigma Aldrich) for 15 minutes. Blots were analyzed and quantified using ImageQuant (Amersham, Little Chalfont, Bucks, UK) software, with the reactive brown image as a control for equal loading and transfer.

### Human muscle satellite cell isolation, proliferation and differentiation

Satellite cells were isolated from *vastus lateralis *muscle biopsies as previously described [47]. Briefly, following removal of fat and connective tissue, the biopsy was digested in a 10 ml buffer containing trypsin and collagenase II for 5+10 minutes. To minimize fibroblast contamination, cells were pre-seeded in a culture dish for 3 hours in F10/HAM, 20% FBS, 1% penicillin/streptomycin (PS), 1% Fungizone. Unattached cells were then removed and seeded into a culture flask, pre-coated with matrigel (BD Biosciences, San Jose, CA, USA). Following 4 days of incubation, the cell culture medium was changed and then every second day thereafter. Cell cultures were expanded and then seeded for proliferation or differentiation. For proliferation, satellite cells were seeded into culture dishes pre-coated with matrigel (BD Biosciences). Cell culture medium was changed to DMEM low glucose, 10% FBS, 1% PS. Cells were allowed to become 75% confluent and then harvested in cell lysis buffer (Cell Signaling Technology). For differentiation, the cell culture medium was changed to DMEM low glucose, 10% FBS, 1% PS and cells were allowed to become completely confluent. When the satellite cells started to change morphology and line-up, the medium was changed to DMEM high glucose, 2% horse serum, 1% PS. At day 5 on low serum, myotubes were formed and harvested in cell lysis buffer (Cell Signaling Technology).

### miRNA target prediction and Gene Ontology analysis

The binding of miRNA to target mRNA occurs between the 'seed' region of the miRNA (nucleotides 2 to 7 of the 5' end of the mature miRNA) and the 3' untranslated region of the mRNA. Gene lists of predicted targets for each modulated miRNA were obtained using TargetScan 4.2 [48]. Several groups have used microarray data to examine the expression changes when a single miRNA changes, and we used the mean absolute expression approach described recently by Arora and Simpson [49] and also the tissue-centric approach described by Sood *et al. *[50] to determine whether we could detect shifts in the average expression of mRNA targets of the muscle-specific miRNAs (miR-1, miR-133a/b and miR-206, collectively known as 'myomirs') in human skeletal muscle. We found no evidence of systematic mRNA changes.

We thus set out to generate a new method of predicting which genes should be altered in the face of multiple changes in miRNA concentration. The development of ranking procedure is described in detail within the results section. We used Gene Ontology analysis [51] to obtain an overview of the functions of predicted gene lists and select protein targets for further evaluation in cell culture and tissue samples. For Gene Ontology analysis we filtered predicted gene target lists using tissue-specific gene expression profiles derived from U133a+2 Affymetrix chip data (n = 118). We also utilized the global muscle transcriptome as the background RNA expression data set, as misleading ontological enrichment *P*-values are yielded when a generic (genome-wide) reference data set is utilized.

## Results

### Global transcription in skeletal muscle is unaltered in type 2 diabetes

Simple hierarchical clustering and scatter plots of 'gene sets' were used to explore the dataset. As can be seen from Figure S2 in Additional file 1 global clustering by subject (n = 118) resulted in a plot that distributed healthy controls (normal glucose tolerance (NGT), black-bar), impaired glucose tolerance (IGT, yellow-bar) and patients (T2D, red-bar) across the data set, with no obvious grouping of subjects and was not dependent on the normalization method (data not shown). The Affymetrix data were then analyzed using SAM [52] and limma in R [53]. No significant differences in individual gene expression were found between the subject groups with either method. To further test this conclusion, we utilized a quantitative correlation analysis approach whereby each individual gene's expression was related to fasting glucose and fasting insulin. This correlation analysis is a logical approach, as the threshold when a patient is diagnosed with T2D is pragmatic, driven by categorization of risk to aid medical treatment. Quantitative SAM analysis produces a FDR for genes that positively and negatively correlated with these two markers of clinical status. A modest number of genes (approximately 50) were found to correlate significantly with fasting glucose (FDR = 5%) and even fewer with insulin levels (approximately 10). However, the correlation coefficients were very modest; gene expression values covered approximately 90% of the range for insulin or glucose and thus can be deemed of limited biological significance (limma based analysis found even fewer genes). Thus, gene chip analysis indicates that T2D and muscle insulin resistance are not associated with global changes in mRNA abundance, despite the sensitivity of the technology [18-20]. We ran two smaller human skeletal muscle studies [20] at the same core-lab and both yielded substantial (1,000 to 3,000) differential expression using the same methods and staff. Given this, and the larger sample size of this diabetes study, and the substantial difference in insulin resistance (Table 1), the lack of global mRNA changes in T2D appears convincing.

### Mitochondrial related transcript abundance is not associated with insulin resistance

**Figure 1:**
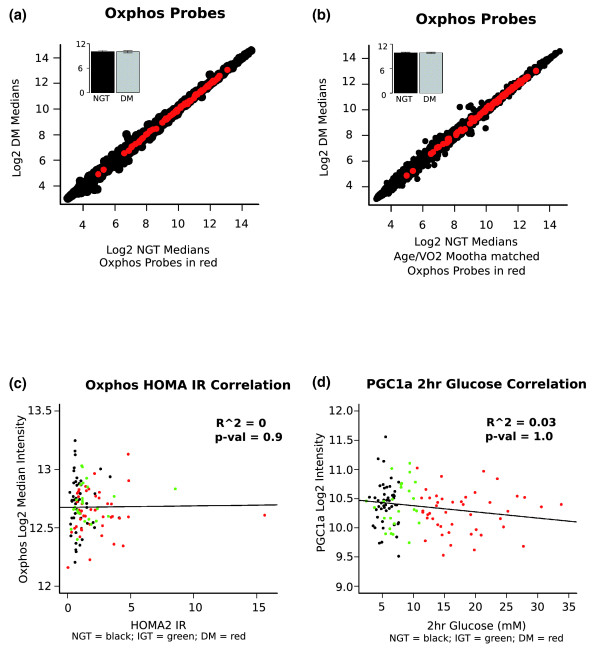
**OXPHOS gene expression and relationship to disease status**. **(a) **Plot of median intensity of OXPHOS probes (red circles) for NGT (n = 47) versus T2D (DM; n = 45) on the background of absent filtered probesets (black circles). The insert shows the mean expression of OXPHOS probesets (± standard error of the mean). **(b) **Plot of median intensity of OXPHOS probes (red circles) for NGT (n = 14) versus T2D (n = 17) on the background of absent filtered probesets (black circles). These subjects have the same physiological characteristics as those in the Mootha *et al. *study [1]. The insert shows the mean expression of OXPHOS probesets (± standard error of the mean). **(c) **Correlation plot for HOMA2 insulin resistance (IR) and MAS 5.0 normalized expression values for the OXPHOS probe sets. Each point represents the median expression for an OXPHOS probe set after filtering the Affymetrix data as described above. The subject groups are represented by colored points: black = normal glucose tolerance; green = impaired glucose tolerance; red = type 2 diabetic. The regression line is shown in black along with the R squared value for goodness of fit and the *P*-value indicating significance of the relationship. **(d) **The linear correlation between 2 hour blood glucose (during oral glucose tolerance test) and PGC-1α expression (n = 118) in skeletal muscle of subjects across the clinical groups NGT (black-dots), IGT (green-dots) and T2D (red-dots) derived from the Affymetrix probe set. The regression line is shown in black along with the R squared value for goodness of fit and the *P*-value indicating significance of the relationship.

Another approach to improve statistical power is to select a small subset of genes on the gene chip for analysis. For example, on the Affymetrix gene chip, >400 genes are annotated as carrying out mitochondrial related functions; this list of genes has been called the 'OXPHOS' gene set [1]. We plotted the expression of the OXPHOS gene set in NGT versus T2D subjects (Figure 1a) and the OXPHOS mRNAs fell on the line of equality, indicating no differential expression. We then investigated if a physiological parameter may explain the difference between our study and that of Mootha. We did this by creating a subgroup of patients (Table S3 in Additional file 1) where the control subjects (n = 14) had a lower BMI and a higher aerobic capacity than the T2D subjects (n = 17) - that is, less well matched - similar to the Mootha *et al. *study. Again, we found no alteration in OXPHOS gene expression (Figure 1b). Furthermore, there is no correlation between OXPHOS gene expression and HOMA1 (Figure 1c) or HOMA2 expression, or between peroxisome proliferator-activated receptor-gamma coactivator-1α (PGC-1α) and plasma glucose concentration (Figure 1d).

We then used a more powerful statistical method, gene set enrichment analysis (GSEA), using both the original [1] and adapted versions of GSEA and their respective 'gene sets' [54]. While we could reproduce the results of Mootha *et al. *using their clinical samples and both methods, when we examined our larger data set, no gene set was enriched (using the original and latest C2.all.v2.5 list). OXPHOS related gene sets (six such lists are included with the program) appeared distributed across the list of enriched genes in control subjects (ranked at positions 8, 14, 57, 66, 370 and 391) and none were statistically significant. Finally, we ran GSEA on the subgroup that re-created the patient characteristics of the Mootha *et al. *study and found that the 'Mootha_VOXPHOS' gene-set had a FDR of 96%. The only remaining distinguishing feature we are aware of, between these studies, is the 3 hour pharmacological insulin infusion protocol utilized by Mootha *et al. *prior to biopsy sampling (see Discussion). Thus, based on analysis of the largest available human muscle T2D array data set, we can conclude that there are no robust changes in protein-coding mRNAs in the skeletal muscle of diabetes patients (although this does not rule out subtle changes in splice variants). The analysis suggests that a post-transcriptional mechanism should exist to regulate the development of insulin resistance in T2D patients, so we tested the hypothesis that altered miRNA expression occurs and in a manner that relates to the development of insulin resistance.

### Analysis of global diabetes-induced changes in skeletal muscle miRNA expression

**Figure 2:**
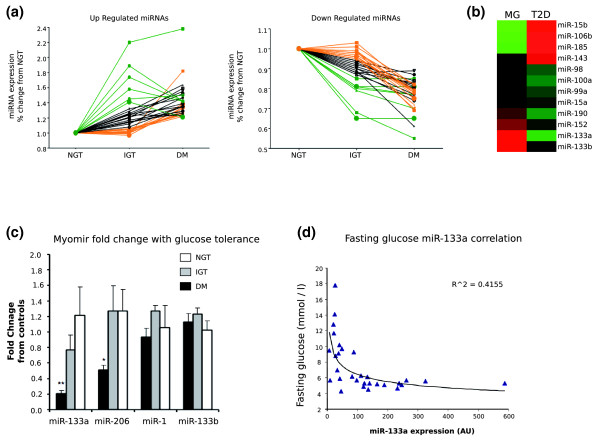
**miRNA expression profile changes in T2D compared with control subjects using the Exiqon chip platform and TaqMan confirmation (FDR <10%)**. **(a) **Data are plotted to show the pattern of change of these significantly up-/down-regulated miRNA. Black lines represent those miRNA that increase/decrease progressively with IGT and T2D (DM), green lines represent miRNAs that are increased/decreased with IGT and then revert with T2D, while orange lines show miRNAs increased/decreased only in the T2D state. **(b) **miRNAs that show the expression profile during myocyte differentiation (cell data derived from Chen *et al. *[55]) is the opposite pattern to that observed in the muscle of patients with T2D (green = down-regulated probe sets, red = up-regulated probe sets; the color range is from -3-fold to +3-fold change). MG refers to the data produced by Chen *et al. *during myogenesis. **(c)**Expression level of miR-1, miR-133a, miR-133b and miR-206 in muscle biopsies from healthy individuals (NGT, n = 10, white bars), individuals with impaired glucose tolerance (IGT, n = 10, grey bars) and individuals with type 2 diabetes (T2D, n = 10, black bars). miR-133a (*P *< 0.001) and miR-206 (*P *= 0.04) were significantly reduced in T2D patients when compared with expression levels in healthy controls. Data are expressed as fold change from NGT and shown as mean ± standard error. ** *P *< 0.001, * *P *< 0.05. **(d) **Expression level of miR-133a in muscle versus indices of glucose homeostasis in subjects with and without T2D. Expression of miR-133a is positively correlated with fasting glucose, R^2 ^= 0.41 (*P *< 0.001, n = 30). Data are shown as ΔCt levels normalized to RNU48 and plotted versus fasting glucose levels (mmol/L).

We detected approximately 170 human miRNAs in skeletal muscle tissue, consistent with muscle expressing a large number of miRNA species. Twenty-nine were significantly up-regulated by >1.3-fold (FDR <10%), while 33 were down-regulated by >1.3-fold (FDR <10%) in T2D (Additional file 2). Taking the miRNAs that were differentially expressed in patients with T2D, we then plotted their expression and included the impaired glucose tolerance samples (Figure 2a). It was clearly evident that approximately 15% of up-regulated and approximately 15% of down-regulated miRNAs were altered early in the disease process, while many changed progressively and a substantial minority were found to be altered only once the patients had diabetes (Figure 2a). By cross-referencing [18] gene chip data sets we identified that 11 from 61 miRNAs demonstrate a pattern of change in expression (Figure 2b) that was the exact opposite of that observed during muscle differentiation [55]. As far as we are aware the only study of myocyte differentiation, in the context of diabetes, derives from streptozotocin-diabetic rats, where primary muscle from diabetic animals fails to robustly fuse to form multinucleated myotubes *in vitro *[56]. Since we observed an inverse relationship between 'muscle development' miRNAs and changes in diabetes, we further investigated the reason for altered expression of the muscle specific miRNAs.

### Muscle-specific mature miRNAs are down-regulated in type 2 diabetes

Mature myomirs were measured in skeletal muscle biopsies from three different groups (Table S1 in Additional file 1; T2D, n = 10; IGT, n = 10; and NGT, n = 10). ANOVA indicated that miR-133a (F = 11.8, *P *< 0.0001) was significantly different between the three groups, miR-206 expression more modestly altered (F = 4.5, *P *= 0.02) and miR-1 and miR-133b were unchanged (Figure 2c). Northern analysis was used to document differences in precursor miR-133 and mature miR-133 abundance. The Northern probe detects both miR-133a and miR-133b due to sequence similarity. The steady state level of pre-miR-133 was very low in human skeletal muscle compared with the signal from the mature miR-133a/b expression transcript (Figure S3 in Additional file 1). This confirms that along with the much lower (>100 times) amplification efficiency [45], miR-133 pre-miRNA cannot contribute to the TaqMan signal.

Skeletal muscle miR-133a expression was reduced by five-fold in T2D (*P *< 0.001). A clear stepwise reduction in mature miR-133a expression was observed across the three clinical groups. We found that expression of miR-133a was associated with fasting glucose and 2 hour glucose tolerance data (R^2 ^= 0.37, *P *< 0.001), with higher fasting glucose levels associated with lower miR-133a expression (Figure 2d). In addition, miR-133a expression was significantly associated with HbA1c, an indicator of long-term glucose homeostasis (R^2 ^= 0.29, *P *< 0.01) and also correlated with HOMA1 (R^2 ^= 0.15, *P *= 0.04). A total of six correlations were carried out and the *P*-values are unadjusted. Subsequently, we checked miR-206, which associated more modestly with these clinical parameters, and miR-1, which did not associate with any of these clinical parameters. Thus, we found that altered miR-133a expression modestly related to important clinical parameters. We then investigated if the altered steady-state level of mature miR-133a was a consequence of failure to produce the primary RNA transcript in the nucleus (Figure S3B in Additional file 1). As the pri-miRNA abundances were unchanged, altered processing or degradation appears responsible for the loss in selective myomir expression rather than altered transcription.

### Detection of miRNA-133a target protein *in vitro *and *in vivo*

There was no change in the mRNA expression of genes that contained myomir target sites (data not shown); thus, miR-133a may only target protein translation rather than mRNA cleavage. Using western blotting, we examined if loss of myomir expression could detectably increase protein targets in a muscle cell model. CDC42 and PTBP1 were selected for study because they ranked highly as targets of miR-133/miR-206 in the TargetScan database and both proteins are relevant for muscle cell differentiation and metabolism [57,58]. Interestingly, reduction in miR-133a using an antagomir (Figure S4A in Additional file 1) had an indirect effect on the other myomirs, such that miR-133b (expected due to sequence similarity) and miR-206 (unexpected) were substantially reduced. This altered expression pattern of mature myomirs was not associated with substantial changes in pri-miRNA expression (Figure S4B in Additional file 1), suggesting some degree of physiological feedback on miRNA maturation during the use of a so-called 'selective' antagomir [59]. Western analysis of CDC42 and PTBP1 demonstrated expected increases (approximately 37% and 20%, respectively) in protein expression following antagomir treatment (Figure S4C in Additional file 1), confirming the suitability of antibodies against them for *in vivo *profiling.

In contrast, analysis of CDC42 and PTBP1 proteins in muscle tissue provided no evidence that these targets were altered *in vivo *(n = 7 to 8 subjects per group; Figure S4D in Additional file 1). Indeed, two recent studies documenting the first global analysis of the relationship between miRNA and the proteome [23,24] found that altered expression of single miRNAs typically had a modest impact on individual protein expression, suggesting to us that the collective changes in many miRNAs may be the most biologically interesting parameter to consider. Thus, we hypothesized that the most likely scenario is that groups of miRNAs work cooperatively *in vivo*, and that physiological regulation of a single muscle protein by a single miRNA may be a rather rare occurrence [60]. It is with this in mind that we set about developing a new ranking system (Figure S1 in Additional file 1) for altered tissue miRNA expression to help define the biochemical consequences of the altered expression of the approximately 60 miRNAs in T2D. Interestingly, our new analysis procedure subsequently identified CDC42 and PTBP1 as being equally targeted by both up- and down-regulated miRNAs (Additional file 2); thus, CDC42 and PTBP1 should not be altered *in vivo *by diabetes (as we demonstrated by western blotting prior to developing our ranking metric).

### A novel weighted context score ranking analysis of global changes in diabetes-induced changes in miRNA expression

Even a modest reduction in protein content can, if within a single canonical pathway, have a strong impact on physiological function. With this in mind, we hypothesized that the main biological consequence of multiple *in vivo *miRNA changes may reflect the collective targeting of multiple members of selected signaling pathways. The collective 'activity' must reflect the observation that both up-regulated and down-regulated miRNA can target the same genes such that the biological impact cannot be assessed using single miRNA-target associations. We devised a ranking system using the conserved target site criteria from the TargetScan database (which is able to significantly enrich a gene population in validated 3' targets [34]) and combined this with our tissue-specific gene and miRNA expression data (Figure S1 in Additional file 1). Evaluation of the ranking procedure was carried out through the identification of statistically enriched and biologically validated gene ontologies and canonical signaling pathways, following adjustment for multiple comparison testing, in the most targeted compared with the least targeted genes. Such an approach was viable using the TargetScan database as we require the context scoring metric as an input for the weighted cumulative context ranking score (wCCS) procedure. An R-script is included (Additional file 2).

Present-marginal-absent call filtering is able to identify, with reasonable sensitivity [42], which mRNAs are expressed in muscle. This list of approximately 20,000 probe sets was cross-referenced with the TargetScan database of miRNA target genes for the 62 T2D miRNAs (approximately 9,000 genes), identifying a total of approximately 4,700 muscle expressed genes with conserved miRNA targets sites for the diabetes-modulated miRNAs. Each target site, on each gene, has a distinct context score relating to the likelihood that a given miRNA will inhibit protein translation or cause mRNA cleavage [25]. Summation of these scores provided us with a range of gene-specific cumulative context scores (CCS) with a distribution shown in Figure S5A in Additional file 1. First quartile ranked mRNAs tended to be expressed at a lower median intensity than fourth quartile targeted genes in control subjects (Figure S5B in Additional file 1), suggesting miRNA-mediated suppression of mRNA abundance or co-evolution of tissue-specific expression. Yet, when tested, we found no association between these miRNA target mRNAs and abundance across the clinical groups (Figure S5C, D in Additional file 1), which is in agreement with our Affymetrix analysis. Indeed, convincing evidence that mRNA cleavage occurs in mammalian cells originates from studies where very large changes in a single miRNA are created by transfection or knock-down and this may not be relevant *in vivo*.

**Figure 3:**
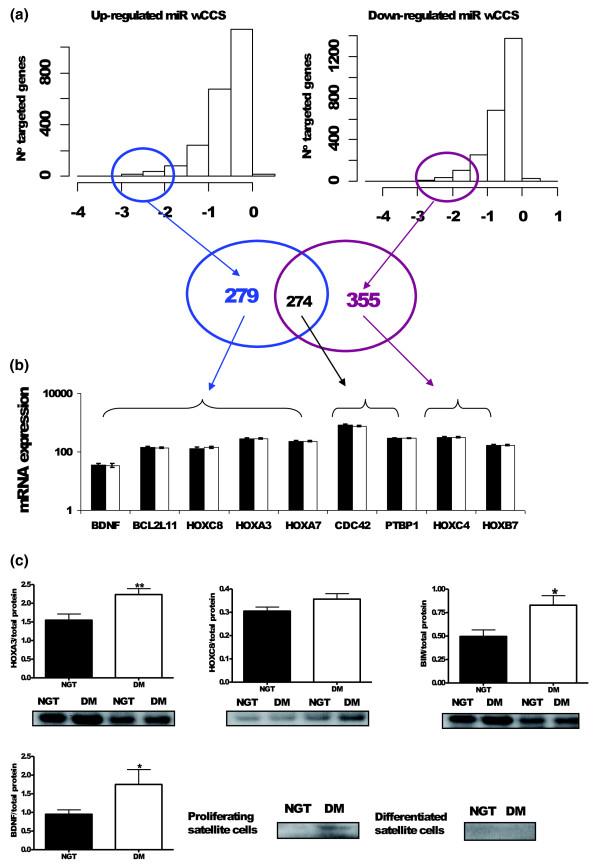
**Generation and validation of a weighted cumulative context score for type 2 diabetes miRNAs**. **(a) **Target genes with a more negative cumulative context score (CCS) are, on average, expressed at a lower level than non-targeted genes (Additional file 2). To determine which genes are most targeted when there is a shift in global miRNA expression, the distribution of CCS was adjusted on a gene by gene basis for the magnitude of up-/down-modulation of [miRNA] - wCCS. As can be seen, despite the vast number of potential predicted targets (Figure S5A in Additional file 1), few target genes have highly scoring wCCSs. There were 279 genes in the first quartile predicted to be up-regulated (reduced regulation by miRNAs) and 355 in the first quartile predicted to be down-regulated (increased regulation by miRNAs). The composition of these lists was validated using pathway and ontology analysis **(b)**. Consistent with the global Affymetrix analysis (Figure S2 in Additional file 1) the mRNA of developmental related first quartile wCCS genes was identical between patients and controls. This was true regardless of whether the gene should be up-regulated (*BDNF*, *BCL2L11(BIM)*, *HOXA3*, *HOXC8*, *HOXA7 *and *HOXB7*), down-regulated (*HOXC4*), or unchanged (*CDC42 *and *PTBP1*). This indicates miRNA are operating to block protein translation. Error bars = s.e.m. **(c) **Proteins highly ranked for being up-regulated were selected and protein expression was analyzed in skeletal muscle biopsies from normal glucose tolerant controls (NGT; n = 6) and subjects with T2D (DM; n = 6). From a second set of subjects, satellite cells were isolated from skeletal muscle biopsies from normal glucose tolerant controls (NGT) (n = 5 to 6) and subjects with T2D (DM; n = 5 to 6). The satellite cells were harvested in a proliferative state or as differentiated into myotubes. Protein expression was analyzed by using western blotting and specific antibodies towards the protein targets. HOXA3 (top left) was detected as a 30 kDa band, significantly up-regulated in muscle from subjects with T2D (*P *= 0.006). BCL2L11 (BIM; top middle) was detected as a band around 25 kDa, significantly up-regulated in muscle from subjects with T2D (*P *= 0.014). HOXC8 (top right) was detected as a band around 36 kDa and demonstrated a clear trend for up-regulation (*P *= 0.07). BDNF (bottom) was detected as a band at 14 kDa, up-regulated in proliferating satellite cells derived from subjects with T2D where it is typically expressed (p = 0.014) but was not expressed in differentiated satellite cells or adult muscle. * = *P *value < 0.05; ** = *P *value < 0.001.

We further reasoned that the net effect of the up-regulated (n = 29) and down-regulated (n = 33) miRNAs on a particular gene would be a product of the change in miRNA expression and the CCS. To model this we adjusted each target site context score by the diabetes related changes in miRNA expression to provide a wCCS. The upper quartile of up- and down-regulated diabetes miRNA targeted genes (first quartile wCCS genes) yields two overlapping gene lists, where approximately 270 targets are common to both lists (Figure 3a). We summed the wCCS for the common 270 genes, taking direction of change into account, and for the majority of cases the wCCS for the up-regulated miRNA targets equaled the wCCS for the down-regulated miRNA targets (suggesting we should expect no net impact on protein expression, for example, for PTBP1). However, for approximately 10% of overlapping genes the wCCS was sufficiently strong such that the gene was retained in either the first quartile up- or down-regulated list.

### Validation of the weighted CCS ranking procedure by ontological and pathway analysis

Ontological analysis is complex and for analysis of these wCCS adjusted target lists we combined the two, non-overlapping (Figure 3a) lists to explore the targeted biological processes. We did this using the muscle-specific transcriptome as the background file (use of the entire genome is inappropriate, as the muscle-specific transcriptome is already highly enriched in ontologies). Highly significant enrichment was uniquely found within the first quartile of ranked genes, including metabolic (*P *< 7.4 × 10^-8^), post-translational modification (*P *< 9.7 × 10^-5^) and developmental (*P *< 1.3 × 10^-6^) processes (all Benjamini-Hochberg adjusted). Further analysis, using only the predicted target list as background (to establish if those genes with the highest wCCS contribute to unique biological activities beyond the ontological profile of the entire miRNA mRNA target list) retained tissue development, and more specifically homeobox gene modulation, as a significant feature (FDR <5%). The 4th quartile of conserved wCCS targets did not demonstrate such enrichment (Additional file 2). Given that the mRNA transcriptome was invariant and the proposed biochemistry of skeletal muscle insulin resistance, modulation of post-translational and metabolic processes is a logical finding, while our analysis highlights muscle development, possibly regulation of muscle stem-cell status, as being of potential importance.

Ontological enrichment of a target gene-list provides statistical evidence of distinct biological processes being targeted by the miRNAs that change in human diabetes, but it remains a further challenge to pinpoint the signaling pathways involved in the disease process from these alone. To this end, canonical pathway analysis was used (based on Ingenuity verified interactions) to visualize whether first quartile genes belong to known insulin resistance related processes. We found approximately six significant canonical pathways (Figure S6 in Additional file 1) represented within our first quartile wCCS list; encouragingly, these represent incompletely described diabetes disease pathways. The highest ranked signaling pathway, transforming growth factor-β signaling, is extensively implicated in all aspects of skeletal muscle function [61], while at an individual gene level, the directional changes in ERK1/2 and MEK1/2 are consistent with the emerging mechanism through which saturated fatty acids induce muscle insulin resistance [62] and with decreased IRS-1 (insulin receptor substrate-1) phosphorylation [63] promoting the degradation of IRS-1 [64] and thus impaired insulin action. Furthermore, modulation of glucocorticoid signaling [65-67], cAMP metabolism [68-70] and BDNF activity [71-75] are connected with insulin resistance in humans and various animal models. Thus, the novel tissue-specific wCCS-based analysis of the 62 miRNAs altered in human diabetic muscle correctly identified diabetes-related disease mechanisms, providing support for this new method of functional annotation of *in vivo *global miRNA data sets. The fourth quartile of conserved wCCS targets did not demonstrate any canonical pathway enrichment above the level of chance. We recently produced a parallel miRNA and mRNA profile of adipogenesis. When applying the wCCS we again found ontological enrichment in the first quartile versus fourth quartile ranked genes; >80% of the first quartile genes were not part of the diabetes miR target list and the ontological profile was distinct (data not shown).

### Protein validation of the wCCS method

While the informatic validation of the ranking procedure was encouraging, it was important to provide evidence that protein abundance changes could be correctly predicted. As noted above, the wCCS correctly identified both CDC42 and PTBP1 protein abundance as unchanged and our protein analysis confirmed this. We then examined the mRNA and protein expression of four additional developmental protein targets that were predicted to be up-regulated either in the skeletal muscle tissue (HOXA3, BCL2L11 (also known as BIM1) and HOXC8) or, in the case of BDNF, in the skeletal muscle satellite cells. These targets were selected based on there being an available and functioning antibody, and because they appear near the top of the first quartile of the wCCS gene list. We noted that yet again there were no shifts in mRNA abundance of these target genes (Figure 3b). Convincingly, we demonstrated that HOXA3 and BCL2L11 proteins were up-regulated by approximately 50%, while BDNF was also up-regulated (Figure 3c). HOXC8 expression was altered less markedly but there was a clear trend consistent with the prediction (*P *= 0.07). We also examined the Baek *et al. *[23] database of *in vitro *global protein changes when miRNAs were artificially manipulated in a HeLa cell system. Our wCCS ranking yielded analysis consistent with their protein level changes (Additional file 2). Thus, protein analysis supported the validity of our tissue-specific wCCS ranking approach for interpretation of the consequences of multiple *in vivo *miRNA changes.

## Discussion

The molecular processes contributing to skeletal muscle insulin resistance are incompletely understood [76], while evidence that developmental factors may play a role is accumulating [77]. The present genome-wide RNA analysis presents further evidence that the human skeletal muscle coding transcriptome in T2D is indistinguishable from that of control subjects. In contrast, miRNA profiling, coupled with the wCCS analysis method, indicates approximately one-third of muscle-expressed miRNAs are altered in diabetes and that collectively these miRNAs target established diabetes-related signaling pathways and highlight a potential role for developmental genes. This included BDNF, which was only expressed in satellite cells and this may be disease specific as it has been found to be unaltered by physical activity status in humans or rodents [18,73]. A seventh protein (LIF) was validated very recently in our lab. However, wider protein level validation of the wCCS approach will require large scale sensitive proteomics, and this is not an easy option with small human clinical samples at this time. Meanwhile, targeted protein profiling of highly ranked proteins identified by our method is a viable alternative for studying miRNA regulated protein networks. Establishment of additional parallel coding and non-coding transcriptome data sets, where multiple miRNA families are simultaneously altered by disease or physiological stimuli, will provide opportunity to further refine the wCCS approach.

### The invariant type 2 diabetes skeletal muscle mRNA transcriptome: experimental design considerations

A limitation of microarray technology is that it does not provide data on possible protein level changes. Nevertheless, if one wants to establish system-wide changes - on the understanding that complex phenotypes involve differential regulation of gene networks, not just individual genes - then microarrays are currently the systems biology tool of choice. In contrast to the unchanged global transcriptome in insulin resistant skeletal muscle, there are several observations that the expression of individual mRNA transcripts display altered expression in the skeletal muscle of patients with T2D on a gene-by-gene basis. However, such changes [78] do not correlate with disease severity and often are not reproducible in larger samples [79]. Using an appropriately matched cohort approximately ten times the size of the Patti *et al. *study [2], we establish that the T2D global muscle coding-RNA transcriptome is invariant, while our subgroup analysis, designed to be comparable with Mootha *et al. *[1], demonstrates that their observation of a reduced OXPHOS gene set in T2D patients appears to reflect the acute differential response to pharmacological levels of insulin [80] in their control subjects, or some other confounding drug treatment in their diabetes patients (for example, statin therapy). This conclusion is in agreement with recent physiological studies [11,81,82] where no intrinsic defect in mitochondrial biochemical function was found in the skeletal muscle of T2D subjects.

Despite this major difference in study interpretation and conclusion, all human microarray studies examining insulin resistance in skeletal muscle paint a remarkably similar picture - one of no striking change in protein coding mRNA abundance. In the Patti *et al. *study [2], muscle samples from a small group of subjects of Mexican-American ethnicity were studied using the Affymetrix HuGeneFL array platform, representing only 15% of the RNA transcriptome, and no significant differences were found. A gene-by-gene qPCR approach was also used, yielding evidence for reduced transcriptional regulators of OXPHOS gene expression [2]. However, as oxidative metabolism proteins can be altered with physical inactivity [15], and a very large difference in demographics existed between the groups [2], then the observation made probably does not reflect diabetes. Another problem with the study by Patti *et al. *[2] was that patients were taken off their medication only 48 hours prior to obtaining the muscle biopsy. In the present study we ensured patients with T2D ceased taking their hypoglycemic medication for 1 week prior to clinical measurements and muscle biopsy. Interestingly, short-term and long-term measures of glucose control - fasting glucose and HbA1c - remained highly correlated (R^2 ^= 0.71) in our study, suggesting that after being treated for a number of years, drug therapy was no longer providing a substantial influence on hyperglycemia [83]. This discussion highlights the possibility that protein signaling changes previously ascribed to the insulin resistance disease process [84] may in fact be a refractory response to pharmaceutical medication and hence represent an artifact of study design.

Mootha *et al. *[1] studied a group of older diabetes subjects (approximately 66 years) using a microarray platform that provides greater coverage of the transcriptome (approximately 20,000 sequences). The authors applied a now robust statistical approach [54] and presented evidence that there was a statistically significant down-regulation of a group of genes involved in oxidative metabolism (OXPHOS) in skeletal muscle of T2D subjects, and claimed that this 'gene set' reflected reduced PGC-1α activity. In the present, much larger analysis we did not identify any correlation between glucose or insulin levels and any gene set including OXPHOS or PGC-1α. To examine the discrepancy between our data set and the Mootha *et al. *study [1], we ran GSEA on a subgroup of our patients that closely approximated the demographics of their study. Hence, the only difference between the two studies should be the 3-hour hyperinsulinemia exposure prior to biopsy sampling in the Mootha *et al. *study. In our subjects, the OXPHOS gene set was ranked the least enriched gene set in the NGT subjects, supporting the idea that obtaining the biopsy samples after a period of pharmacological hyperinsulinemia created an acute change in OXPHOS genes as T2D patients will respond differently to pharmacological levels of insulin infusion compared to control subjects [3]. Thus, although substantial loss of mitochondrial function can cause metabolic dysfunction and muscle insulin resistance or diabetes [5], this is not synonymous with evidence that OXPHOS defects are a causal or primary defect in T2D and we cannot demonstrate that such a defect exists in the skeletal muscle of diabetes patients. Further, the major determinants of skeletal muscle mitochondrial status - physical activity and physical fitness [85] - were not controlled for in any study and thus the OXPHOS-diabetes disease association should be considered unreliable.

### Coordinated alteration in human skeletal muscle miRNA expression relates to insulin resistance in type 2 diabetes

We provide new evidence that disrupted miRNA expression may have relevance for insulin resistant skeletal muscle. Firstly, one-third of miRNAs robustly expressed in muscle (62 out of 171) have altered expression in diabetes patients and a subset of these is altered early in disease where patients remain untreated (Figure 2a). Secondly, we demonstrate that the highest ranked wCCS genes belonged to relevant biochemical processes, namely post-translational modification and metabolic pathways. Further, the genes ranked as being targeted most strongly by the collective net changes in miRNA expression target approximately six significant canonical signaling pathways, five of which are described as related to insulin resistance or muscle metabolism [65-75]. This level of statistical evidence is robust, especially when one considers the fourth quartile ranked genes demonstrated no such associations.

Several miRNAs are highly regulated *in vivo *and *in vitro *during muscle development and these regulate the muscle differential expression process [55]. Most studied are miR-133, miR-206 and miR-1, which are all induced during differentiation of myoblasts into myotubes [28]. We were able to demonstrate using a separate detection system that altered myomir expression varies with disease severity and that gene-chip expression of a subgroup of miRNAs (10 out of 11) was regulated in a manner diametrically opposite that observed during muscle differentiation. Over-expression of miR-1 [55] or miR-206 [86] in mouse myoblasts accelerates differentiation into myotubes whereas over-expression of miR-133 promotes proliferation [55]. *In vivo *the expression of these miRNAs can vary as miR-1 and miR-133a decrease 50% in response to muscle hypertrophy in mice following 7 days of loading [87]. As discussed below, and implicit in the successful identification of diabetes disease processes using the wCCS ranking approach and *in vivo *miRNA profiling, it is the combinatorial nature of miRNA action *in vivo *that seems to be most relevant. To this end we have been able to call the protein expression differences correctly (seven from seven) between controls and TD2 subjects using the wCCS ranking approach, and in doing so expand the evidence base for the involvement of developmental genes in muscle insulin resistance. These observations indicate that we have made progress in addressing a major challenge in the miRNA field, namely that of interpretation of biological consequences of *in vivo *multiple miRNA modulation [23].

Using the myomir family as an example, we attempted to establish why we observed changes in mature miRNA abundance. Current understanding of miRNA biogenesis and processing is primarily based on *in vitro *and genetic studies in lower organisms [88]. Mature miRNAs are derived from a longer primary transcript - approximately 1 to 3 kb transcribed by RNA polymerase II [89] - that are then processed in the nucleus by Drosha to form an approximately 70- to 80-nucleotide precursor miRNA [90]. This pre-miRNA is exported to the cytoplasm via Exportin 5 [91] where Dicer cleaves the pre-miRNA to leave a 20- to 22-nucleotide mature miRNA that is incorporated into a waiting RISC complex, where it can bind complementary target mRNAs and suppress translation of multiple mRNAs. Many miRNAs are transcribed as a 'cluster' from a single genomic region and it has been stated that for the myomirs, each should be co-transcribed and co-expressed. However, evidence of distinct binding proteins that modulate processing of pri-miRNA to mature miRNA [92] has emerged and we clearly demonstrate that expression of miR-1 and miR-133a are not co-regulated *in vivo *in human skeletal muscle. This suggests that either processing of the pri-miR-133a or stability of mature miR-133a is altered in T2D. Regulation of miRNA production, post-transcriptionally, is proving to be potentially important for determining stem cell differentiation [93,94] while the protein or signaling factors that inhibit miR-133a production in T2D remain to be determined, this process clearly has the potential to alter muscle differentiation [28].

### Human skeletal muscle insulin resistance and developmental genes

Given the chronic nature of skeletal muscle insulin resistance in diabetes and the role of satellite cells in maintaining long-term physiological function [95], it is surprising that so little is known about muscle stem cell status in T2D. So we were particularly interested in the idea that satellite cell function may be altered in TD2 [73]. Our analysis indicated that modulated miRNAs collectively target developmental processes (*P *< 1.3 × 10^-6^) and thus we speculate that at least part of the disease process occurs within the skeletal muscle stem cells (satellite cells). Disrupted muscle repair would be consistent with the involvement of BDNF expression inhibiting myogenesis [96] and we demonstrated that BDNF is elevated in proliferating satellite cells from diabetes patients (Figure 3c). Interestingly, BDNF mRNA expression is not altered by endurance training [18] and additional RT-qPCR on this material (n = 24, data not shown) found it barely detectable in adult muscle tissue. Indeed, BDNF was only reliably detectable in activated muscle satellite cells. Studies on muscle damage in chemically induced diabetes models show impaired recovery [73], while this interesting link between diabetes, BDNF and muscle recovery remains to be studied in humans.

In support of our focus on developmental genes, pathway analysis of recent genome-wide association studies, which so far have yielded few T2D candidate genes, provided an integrated interpretation of the highest ranked risk genes for T2D [97]. This analysis found that lipid metabolism and developmental genes were significantly over-represented in the upper ranked genes of the T2D genome-wide association studies, an observation based on thousands of samples, and one strongly consistent with the present independent analysis. Combined, we believe this presents strong evidence that developmental genes may play a role in setting or regulating the long-term responses of skeletal muscle to diabetes.

## Conclusions

In the present analysis, we provide robust evidence that combining multiple single-gene predictions produced a set of targets that could be validated at several levels. Indeed, we have so far found the method to be 100% accurate. However, there are a number of additional theoretical considerations that need to be mentioned, as the wCCS method currently does not include potentially important information. Firstly, we did not integrate the target site multiplicative effect [33] due to a lack of information on the synergy between the proximity of heterogeneous miRNA target sites and protein translational block. Thus, as lower ranked protein targets are considered, the precision of the method may decline. Nor did we integrate absolute miRNA abundance data. Thus, we did not distinguish between changes in high abundance and low abundance miRNAs. The main reason for this omission is that we can not accurately compare miRNA abundance across probes on a microarray, as each probe produces linear detection of single miRNA abundance and the signal is not designed to be compared across detection probes. Nevertheless, given the enormous range of probe intensities, it is likely that some changes do represent much larger absolute alterations in miRNA concentration than others. Thus, it may be possible to further refine the interpretation of coordinated *in vivo *changes in miRNA expression if we adjust the wCCS score by miRNA absolute concentration. One needs to do this with some caution as the precise 'potency' of a given miRNA, as well as subcellular compartmentalization, ensures that such a calculation is unlikely to be a simple linear one.

The new ranking strategy detects relevant biology without bias relating to protein isolation or chemistry and thus can aid pathway mining where clinical biopsy size prevents global proteomics. The present analysis indicates that collective miRNA changes *in vivo *should be taken into account. Technically, it would be challenging to mimic this in cells as the simultaneous knock-down of 33 miRNA combined with over-expression of 29 up-regulated miRNAs, all at the correct dosage, is intractable and would be of questionable physiological relevance in a cell culture system. In conclusion, we provide the first global RNA profile of human skeletal muscle insulin resistance and demonstrate a remarkably invariant mRNA landscape. We present a new method for interpretation of multiple miRNA changes *in vivo*, analysis that extends the evidence that developmental genes play a role in metabolic disease [97,98]. miRNAs can be robustly detected in minute amounts of RNA, collected by pain-free micro-needle sampling, such that we believe they represent plausible biomarkers of muscle status, and may be useful for monitoring pharmacodynamics and early-stage efficacy during larger-scale diabetes intervention trials

## Abbreviations

BDNF: Brain-derived neurotrophic factor; BMI: body mass index; BSA: bovine serum albumin; CCS: cumulative context score; DMEM: Dulbecco's modified Eagle's medium; FBS: fetal bovine serum; FDR: false discovery rate; GSEA: gene set enrichment analysis; HOMA: homeostatic model assessment; IGT: impaired glucose tolerance; LNA: locked nucleic acid; MAS: Microarray Suite; miRNA: microRNA; NGT: normal glucose tolerance; OXPHOS: oxidative phosphorylation; PGC-1α: peroxisome proliferator-activated receptor-gamma coactivator-1α; PS: penicillin/streptomycin; PTBP1: Polypyrimidine tract-binding protein 1; qPCR: quantitative real-time PCR; RT: reverse transcription; SAM: significance analysis of microarray; T2D: type 2 diabetes; TBST: Tris-buffered saline with Tween20; wCCS: weighted cumulative context ranking score.

## Competing interests

This study was supported by an Affymetrix Translational Medicine award (JT). This reduced the cost of the gene-chip screening. They had no role in study design, data collection and analysis, decision to publish, or preparation of the manuscript.

## Authors' contributions

JAT conceived the idea for the project (coding and non-coding gene-arrays to study insulin resistance) in 2006. JAT, PK and IJG developed and implemented the miRNA analysis methods. JAT, PK, CS, KR, JR, CW, JB, and GH refined these ideas and implemented the full up study analysis. BKP, ARN and CPF carried out the clinical data collection. JR, JB, KR, CS, IJG and PK carried out the molecular analysis. JAT and IJG carried out the informatics analysis. JAT drafted the manuscript. JAT, IJG, CS, PK, ARN, JR, CPF, KR, JB, CW, GH and BKP edited the manuscript.

## Supplementary Material

Additional file 1 Figures S1 to S6, Tables S1 to S4 and supplementary Results and Discussion. Click here for file

Additional file 2 miRNA lists and subject demographics. Click here for file
