# Supplementary material for: Integration of microRNA changes in vivo identifies novel molecular features of muscle insulin resistance in type 2 diabetes
Source: Genome Med. 2010 Feb 1;2(2):9. doi: 10.1186/gm130 (PMC2847700; doi:10.1186/gm130)

**Figure S1 - In vivo miRNA target prediction and Ontology analysis flow chart**

This is a representative outline of the wCCS strategy. The binding of miRNA to target mRNA occurs between the “seed” region of the miRNA (nucleotide 2-7 of the 5`end of the mature miRNA) and the 3`UTR of the mRNA. Genes predicted to be regulated by specific miRNAs were obtained using Targetscan 4.2 [1]. Several groups have used microarray data to examine the expression of miRNA targets across tissues, and we used the mean absolute expression approach described recently by Arora and Simpson [2] and also the tissue-centric approach described by Sood *et al.* [3] to determine whether we could detect shifts in the average expression of miRNA targets in human skeletal muscle when expression of a particular miRNA was altered. For miRNAs differentially expressed we also used Gene Ontology (GO) analysis [4] to obtain an overview of the functions of genes predicted or known to be targeted by these specific miRNAs. Importantly we carried out pre-filtering of the predicted gene target lists using tissue specific gene expression profiles derived from U133a+2 Affymetrix chip data (n=118) to improve the precision of the ontology profiles, with the reference list representing all muscle expressed mRNAs. DAVID (http://david.abcc.ncifcrf.gov/) was used to find enriched GO categories.

**Figure S2**. **Heatmap of RMA normalized data, filtered for MAS 5.0 present-called probesets from all patient groups (n=118).** Subjects are in rows and the 1000 most variable (largest SD) probesets are arranged in columns. The subjects’ clinical status is denoted by the side bar; black=normal glucose tolerance (NGT, n=47), yellow=impaired glucose tolerance (IGT, n=26), red=type 2 diabetes (T2D, n=45). Hybridization, washing, staining and scanning of the arrays were performed according to manufacturer’s instructions (Affymetrix, Inc. http://www.affymetrix.com/). We utilized the Affymetrix U133+2 array platform and 15 µg of cRNA was loaded onto each chip. All array data were normalized using the Microarray Suite version 5.0 (MAS 5.0) algorithm to a global scaling intensity of 100. Arrays were examined using hierarchical clustering to identify outliers prior to statistical analysis, in addition to the standard quality assessments including scaling factors and NUSE plot. No array included in this analysis failed these standard quality assurance procedures. We relied on several statistical approaches to analyze the data with and without pre-filtering of gene lists. The microarray data were subjected to global normalization using the Robust Multi-Array Average expression measure (RMA) in the Bioconductor suite (http://www.bioconductor.org) and analyses were compared in parallel with MAS 5.0 based normalization. Using the MAS 5.0 generated present-absent calls can also improve the sensitivity of the differential gene expression analysis [5] as it increases the statistical power of the analysis. We chose to remove probe sets that were declared ‘absent' across all chips in the study. These normalized data were utilized for subsequent analysis described in the results section. The CEL files have been deposited at GEO under reference number GSE18732 and patient phenotype data has also been made available at the same location.

**Figure S3. Transcript analysis of the muscle specific miRNAs – the myomirs - and their precursor RNA (pri-miRNAs). (a)** Expression level of miR-1, miR-133a, miR-133b and miR-206 in muscle biopsies from healthy individuals (NGT, n=10, white-bars), individuals with impaired glucose tolerance (IGT, n=10, grey-bars) and individuals with type 2 diabetes (T2D, n=10, black-bars). MiR-133a (p<0.001) and miR-206 (p=0.04) were significantly reduced in T2D patients when compared with expression levels in healthy controls. Data are expressed as raw CT values, adjusted by RNA loading (18S/RNU48) shown as mean±SE. A higher CT value reflects a lower gene expression **denotes p<0.001, *denotes p<0.05. Northern blot of miR-133 was carried out using RNA pooled from several subjects within each group. Northern blot demonstrates that miR-133a/b is highly expressed in muscle while expression of pre-miR-133a/b is negligible thus indicating that the TaqMan assay almost entirely reflects the mature miRNA abundance. **(b)** PCR amplification of the precursor miR-1 and miR-133a on human chromosome 18 and chromosome 20. Primers were designed to amplify the genomic region (pri-miRNA) upstream and downstream of the predicted pre-miRNA stem-loops to determine the expression of the primary transcripts for miR-1 and miR-133a. Primers are labeled A1-A4 and the sequences can be found in table S4. Pri-mir-1-1, pri-mir-1-2, pri-mir-133a-2 and pri-mir-133a-1 primers were tested across a range of cDNA dilutions to determine primer efficiency. With similar primer efficiencies the absolute abundance of each pri-miRNA transcript can be compared with neighboring transcripts. The location of the mature miRNA is identified as the red sequence on the pre-miRNA stem-loop. RT-PCR cycle thresholds in T2D, IGT and NGT groups for pri-mir-1-1-R (R = region), pri-mir-133a-2-R, pri-mir-1-2-R and pri-mir-133a-1-R as shown. Pri-mir-133a-2-R is the most abundant transcript and pri-mir-133a-1-R is the least. NGT (n=10, white-bars), IGT (n=10, grey-bars) and T2D patients (n=10, black-bars). Expression level of the pri-mir-1-1, pri-mir-133a-2, pri-mir-1-2 and pri-mir-133a-1 in muscle biopsies from NGT (white-bars), IGT (grey-bars) and T2D patients (black-bars) normalized to 18S. There were no significant differences in pri-miRNA-R transcripts between NGT, IGT and T2D patients and thus no difference in the primary precursor for miRNA production.

**Figure S4.** **Myomir single target protein analysis in cells and in patient tissue.** (**A)** Northern blot of antagomir (100nM) induced knockdown of miR-133a in C2C12 myoblasts 48 hrs after transfection. RT-qPCR was used to confirm changes in muscle specific miRNA expression following antagomir knockdown of miR-133a in C2C12 myoblasts (n=2-3 transfections). It was noted, unsurprisingly, that the single targeting of a miRNA using a selective antagomir tool yields a number of additional alterations. miR-133b was substantially knocked down, reflecting close sequence similarity to miR-133a. The unrelated sequences miR-206 and miR-1 were also changed, probably reflecting the biological consequences of loss of the miR-133 family. **(B)** Note that determination of pri-miRNA levels using rt-QPCR, demonstrated that these interactions appear to occur at the post-transcriptional level, as neither pri-1, pri-206 nor pri-133b levels were altered. **(C)** Using Western blotting, changes in miR-133a target protein expression (CDC42 and PTBP1) following knockdown of miR-133a using antagomirs (ATG) in C2C12 myoblasts (miR-133a ATG: myoblasts transfected with 100nM miR-133a ATG; Scrambled ATG: myoblasts transfected with a scrambled ASO; none: non-transfected myoblasts). **(D)** Western analysis of CDC42 and PTBP1 protein expression in skeletal muscle of normal glucose tolerance subjects (NGT) and type 2 diabetic subjects (T2D) indicated that no difference was found. Note, that following the development of the target ranking procedure, this lack of change in protein for CDC42 and PTBP1 was to be expected as both proteins were equally targeted by the up and down regulated miRNAs *in vivo*.

**Figure S5. Distribution of cumulative context scores for miRNA targeted mRNA levels in muscle and for those mRNA targeted by miRNAs altered in T2D.** **(a)** Distribution of the cumulative context score for all the genes which have a binding site for the 170 miRNAs expressed in muscle. Genes with scores in the first (1Q) and fourth quartile (4Q) of this distribution were picked for further analysis. **(b)** Median expression of all present called genes on the HGU133plus2 array (black bar), that is all mRNAs reliably detected by the gene array platform. For those genes (1Q genes, grey bar) with the most muscle miRNA binding sites (or best context scores, more negative values) the median expression of those mRNAs were substantially lower than those expressed genes with the least probability of being regulated by the 170 muscle specific miRNAs (4Q genes, white bar). (**c**) Despite this association between muscle miRNAs and global mRNA expression, there was no further interaction noted when the genes most targeted by the 62 diabetes regulated miRNAs were compared with those genes with the lowest weighted cumulative context scores. It would appear that while there is some general connection between [miRNA] and [mRNA], the biological impact of disease regulated miRNA is not via altered mRNA abundance in human muscle diabetes or insulin resistance.

**Figure S6**. Illustrations of biological pathways that the altered miRNA target, corrected for multiple testing. (A) Canonical pathway analysis using Ingenuity software (www.ingenuity.com) demonstrated that the 1Q genes were clearly enriched for membership of 6 canonical pathways (TGF-β signalling, RAR activation, cAMP mediated signalling, BMP signalling, glucocorticoid receptor signalling and neurotrophin / TRK signalling (e.g. BDNF)) with all Benjamini-Hochberg corrected p-values below 0.05 (yellow dashed line). The background file used for testing enrichment was all genes expressed in muscle from the Affymetrix analysis. (B) In three example pathways, blue indicates the gene is targeted by the cumulative impact of the up-regulated microRNAs and hence protein expression is expected to be decreased, while genes coloured red are targeted by the cumulative change in down-regulated microRNAs and hence protein expression would be expected to be up-regulated.

**Supplemental Tables**

**Table S1 - Clinical characteristics of miRNA profiling group**

|  | **T2D**  **(n=10)** | **IGT**  **(n=10)** | **NGT**  **(n=10)** |
| --- | --- | --- | --- |
| Age | 60.4 ± 14 | 60.2 ± 7.1 | 60.3 ± 7.5 |
| BMI | 26.6 ± 1.9 | 26.3 ± 1.7 | 25.9 ± 1.9 |
| VO_2max_ | 28.8 ± 8.6 | 29.4 ± 6.9 | 28.4 ± 6.0 |
| Fasting glucose | 11.3 ± 2.9* | 5.9 ± 0.5# | 5.0 ± 0.4 |
| 2-h glucose tolerance | 21.1 ± 5.1* | 7.5 ± 1.8# | 5.1 ± 1.6 |
| HbA1c | 8.3 ± 1.3* | 5.8 ± 0.2$ | 5.6 ± 0.3 |

Data are mean ± standard deviation. BMI is body mass index; VO_2max_ is ml/kg/min; Fasting glucose and 2-h glucose tolerance is mmol/L; HbA1c is % glycosylated haemoglobin. * denotes p<0.001 when compared with either NGT or IGT; # denotes p<0.01 and $ denotes p<0.05 when compared with the NGT group.

**Table S2**

| **Target** | **Identifier** | **Primers, sequences or probes** |
| --- | --- | --- |
| Pri-hsa-mir-1-1 | ENSG00000199017 | 5’-caggcgctcgagactttct-3’ (forward)  5’-tcacacactcacacgatcca-3’ (reverse) |
| Pri-hsa-mir-133a-2 | ENSG00000207764 | 5’-tctatcctatggctcacaaaagc-3’ (forward)  5’-ctcactcacgggtggaaac-3’ (reverse) |
| Pri-hsa-mir-133a-1 | ENSG00000207786 | 5’-aaatgtactttctgtgactgaggtgt-3’ (forward)  5’-ctgtgggcaaaaggagacat-3’ (reverse) |
| Pri-hsa-mir-1-2 | ENSG00000207694 | 5’-aagttgttagctgtaaaaacatgaaa-3’ (forward)  5’-ttcgataaattagtctctgcaaatg-3’ (reverse) |
| hsa-miR-1 | MIMAT0000465 | 5’-uggaauguaaagaaguaugua-3’  Taqman® MicroRNA assay Cat# 4373161 |
| hsa-miR-133a | MIMAT0001475 | 5’-uugguccccuucaaccagcugu-3’  Taqman® MicroRNA assay Cat# 4373142 |
| hsa-miR-133b | MIMAT0000770 | 5’-uugguccccuucaaccagcua-3’  Taqman® MicroRNA assay Cat# 4373172 |
| hsa-miR-206 | MIMAT0000879 | 5’-uggaauguaaggaagugugugg-3’  Taqman® MicroRNA assay Cat# 4373092 |
| RNU48 | NR_002745 | 5’-gaugaccccagguaacucugagugug  ucgcugaugccaucaccgcagcgcucugacc-3’  Taqman® MicroRNA assay Cat# 4373383 |
| hsa-miR-133a  hsa-miR-133b | MIMAT0001475 MIMAT0000770 | 5’-agcugguugaaggggaccaaa-3’  (Northern probe) |

All primer sequences for pri-miRNA transcripts were obtained from Invitrogen, UK. Location of pri-miRNA transcripts is shown in Figure S1A. A1=pri-miR-1-1, A2=pri-miR-133a-2, A3=pri-miR-133a-1, A4=pri-miR-1-2. Muscle-specific mature miRNA were profiled using Taqman® MicroRNA Assays, reagents were obtained from Applied Biosystems. The probe for the Northern blot was designed to detect pre-miR-133a/b and mature miR-133a/b. Pre-miRNA are denoted by Ensembl identifiers (ENSG) and mature miRNA are denoted by miRBase identifiers (MIMA).

**Table S3 - Clinical characteristics of our group matching the Mootha *et al.* study** [6]

|  | **Present Study** | | **Mootha *et al* Study** | |
| --- | --- | --- | --- | --- |
|  | **T2D**  **(n=18)** | **NGT**  **(n=14)** | **T2D**  **(n=18)** | **NGT**  **(n=14)** |
| Age | 63.2 ± 5.3 | 61.6 ± 4.4 | 65.5 ± 1.8 | 66.1 ± 1.0 |
| BMI | 31.2 ± 4.4 | 26.6 ± 3.9 | 27.3 ± 4.0 | 23.6 ± 3.4 |
| VO_2max_ | 25.9 ± 7.8 | 30.5 ± 10.9 | 24.3 ± 5.6 | 32.1 ± 5.5 |
| Fasting glucose | 10.1 ± 3.3 | 4.9 ± 0.4 | 7.8 ± 2.3 | 4.7 ± 0.5 |
| 2-h glucose tolerance | 18.6 ± 5.2 | 5.4 ± 1.3 | 14.9 ± 4.0 | 6.6 ± 0.9 |
| HbA1c | 7.4 ± 1.2 | 5.6 ± 0.2 |  |  |

Data are mean ± standard deviation. BMI is body mass index; VO_2max_ is ml/kg/min; Fasting glucose and 2-h glucose tolerance is mmol/L; HbA1c is % glycosylated haemoglobin.

**Table S4**

| **Target** | **Identifier** | **Primers, sequences or probes** |
| --- | --- | --- |
| Pri-mmu-mir-1-1 | ENSMUSG00000070144 | 5’-caggcgctcgagactttct-3’ (forward)  5’-tcacacactcacacgatcca-3’ (reverse) |
| Pri-mmu-mir-133a-2 | **ENSMUSG00000065460** | 5’-tctatcctatggctcacaaaagc-3’ (forward)  5’-ctcactcacgggtggaaac-3’ (reverse) |
| Pri-mmu-mir-133a-1 | **ENSMUSG00000065399** | 5’-aaatgtactttctgtgactgaggtgt-3’ (forward)  5’-ctgtgggcaaaaggagacat-3’ (reverse) |
| Pri-mmu-mir-1-2 | **ENSMUSG00000080662** | 5’-aagttgttagctgtaaaaacatgaaa-3’ (forward)  5’-ttcgataaattagtctctgcaaatg-3’ (reverse) |
| mmu-miR-133a | MIMAT0000145 | 5’-uugguccccuucaaccagcugu-3’  Taqman® MicroRNA assay Cat# 4373142 |
| Sno142 | AF357324 | 5’-gucagugccacgugucugggccacugagaccacaugaugggauugaggaccugaggaa-3’  Taqman® MicroRNA assay Cat# 4380913 |
| mmu-miR-133a/b | MIMAT0000145  MIMAT0000769 | 5’-agcugguugaaggggaccaaa-3’, (northern probe) |
| t-RNA |  | 5’-ugguggcccguacggggaucga-3’, (northern probe) |
| mmu-miR-133a | MIMAT0000145 | miRCURY™ LNA knockdown probe, Exiqon Cat #139460-00 |
| Scrambled-miR |  | miRCURY™ LNA knockdown probe, Exiqon Cat #199002-00 |

All primer sequences for mouse pri-miRNA transcripts were obtained from Invitrogen UK. Location of pri-miRNA transcripts is shown in Figure S1A. B1=pri-miR-1-1, B2=pri-miR-133a-2, B3=pri-miR-133a-1, B4=pri-miR-1-2. Mature miRNA were profiled using Taqman® MicroRNA Assays (Applied Biosystems). The probe for the Northern blot was designed to detect pre-miR-133a/b and mature miR-133a/b. Pre-miRNA are denoted by Ensembl identifiers (ENSG) and mature miRNA are denoted by miRBase identifiers (MIMA).

**Supplementary Results - discussion**

***Mechanism for in vivo loss of mature myomir expression***

During miRNA biogenesis, mature miRNAs are transcribed from longer pri-miRNA transcripts which are then cleaved to form pre-miRNA and later mature miRNA. In mouse, pri-mir-1 and pri-mir-133a are reportedly transcribed together as one primary transcript in muscle [7, 8] and thereafter subject to differential splicing, while notably each miRNA precursor may have its own MEF2/MyoD promoter [8]. To establish whether the down-regulation of mature human miR-133a in T2D patients reflects altered pri-miRNA expression, we profiled the expression of human pri-miRNA *regions* (pri-mir-1-1 and pri-mir-133a-2 along with pri-mir-1-2 and pri-mir-133a-1 amplicons as indicated). While we found no relationship to metabolic status, there were very clear differences in the abundance of the pri-related transcripts (e.g. pri-mir-1-1 appears to be >4 times less abundant than pri-mir-133a-2 despite originating from the same bicistronic RNA molecule) and hence the processing of the individual pri-miRNA transcripts in human skeletal muscle appears distinct. As primer amplification efficiencies were nearly identical across the different transcripts, it is unlikely that differences in primer amplification efficiency account for the variation in pri-miRNA abundance. The RNU48-adjusted Ct values for miR-1, miR-206, miR-133a and miR-133b demonstrate that the latter two are the most abundant (smallest delta cycle threshold (▲Ct) value). Thus, given the pattern of pri-miRNA expression and that mature miR-133a and miR-1 can independently vary *in vivo* this suggests that either miRNA processing or stability are distinctly regulated in diabetes (despite their bicistronic origin), partly reflecting independent promoter regulation (See Figure 2 in Liu *et al.*, [8]). In the present study, substantial loss of miRNA-133a did not reflect loss of production of the pri-miRNA and thus processing or stability of the miRNA must be altered by diabetes.

1. Lewis BP, Shih IH, Jones-Rhoades MW, Bartel DP, Burge CB: **Prediction of mammalian microRNA targets**. *Cell* 2003, **115**(7):787-798.

2. Arora A, Simpson DA: **Individual mRNA expression profiles reveal the effects of specific microRNAs**. *Genome Biol* 2008, **9**(5):R82.

3. Sood P, Krek A, Zavolan M, Macino G, Rajewsky N: **Cell-type-specific signatures of microRNAs on target mRNA expression**. *Proc Natl Acad Sci U S A* 2006, **103**(8):2746-2751.

4. Ashburner M, Ball CA, Blake JA, Botstein D, Butler H, Cherry JM, Davis AP, Dolinski K, Dwight SS, Eppig JT *et al*: **Gene ontology: tool for the unification of biology. The Gene Ontology Consortium**. *Nat Genet* 2000, **25**(1):25-29.

5. Choe SE, Boutros M, Michelson AM, Church GM, Halfon MS: **Preferred analysis methods for Affymetrix GeneChips revealed by a wholly defined control dataset**. *Genome Biol* 2005, **6**(2):R16.

6. Mootha VK, Lindgren CM, Eriksson KF, Subramanian A, Sihag S, Lehar J, Puigserver P, Carlsson E, Ridderstrale M, Laurila E *et al*: **PGC-1alpha-responsive genes involved in oxidative phosphorylation are coordinately downregulated in human diabetes**. *Nat Genet* 2003, **34**(3):267-273.

7. Chen JF, Mandel EM, Thomson JM, Wu Q, Callis TE, Hammond SM, Conlon FL, Wang DZ: **The role of microRNA-1 and microRNA-133 in skeletal muscle proliferation and differentiation**. *Nat Genet* 2006, **38**(2):228-233.

8. Liu N, Williams AH, Kim Y, McAnally J, Bezprozvannaya S, Sutherland LB, Richardson JA, Bassel-Duby R, Olson EN: **An intragenic MEF2-dependent enhancer directs muscle-specific expression of microRNAs 1 and 133**. *Proc Natl Acad Sci U S A* 2007, **104**(52):20844-20849.


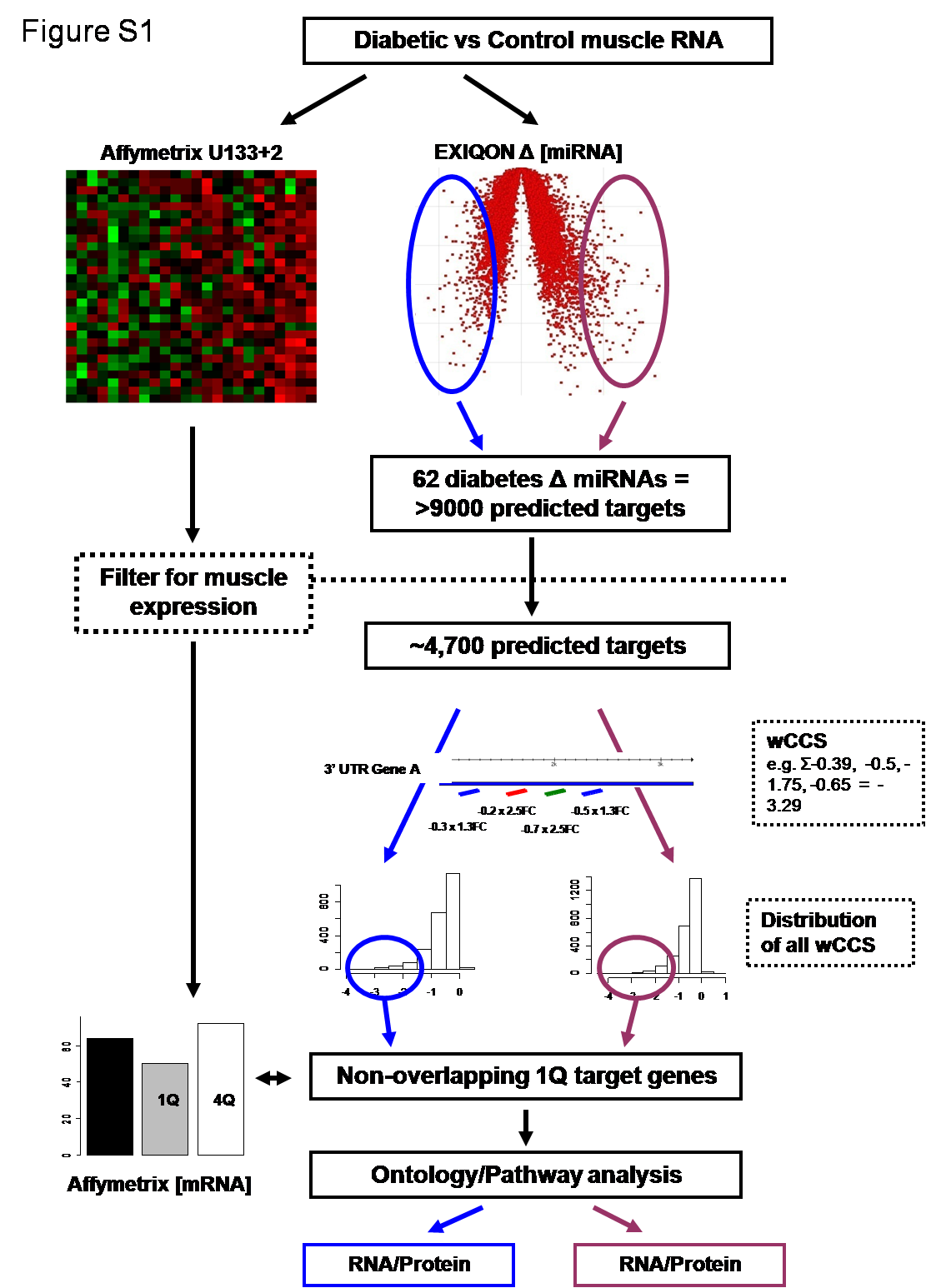


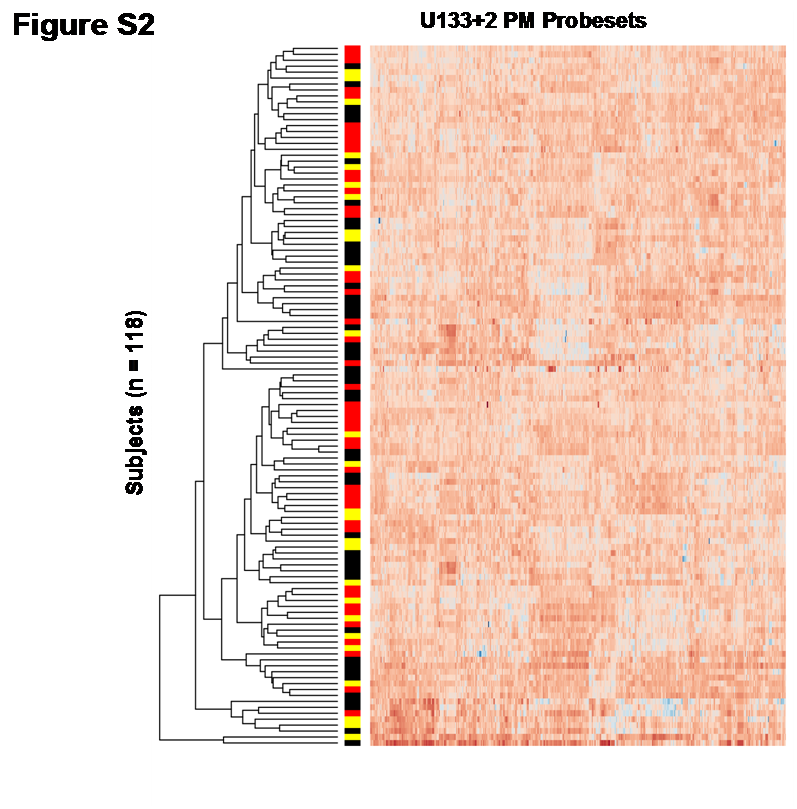


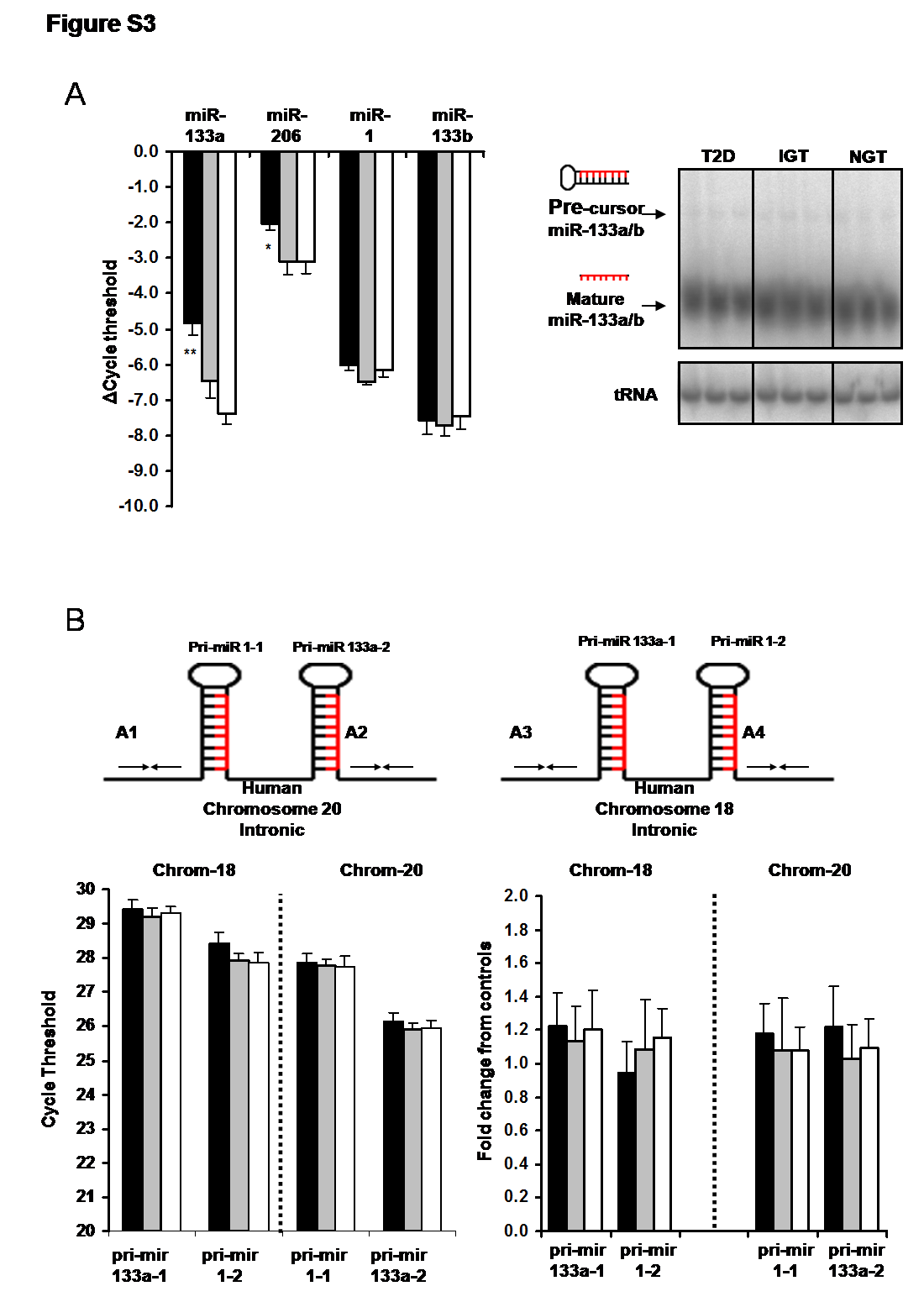


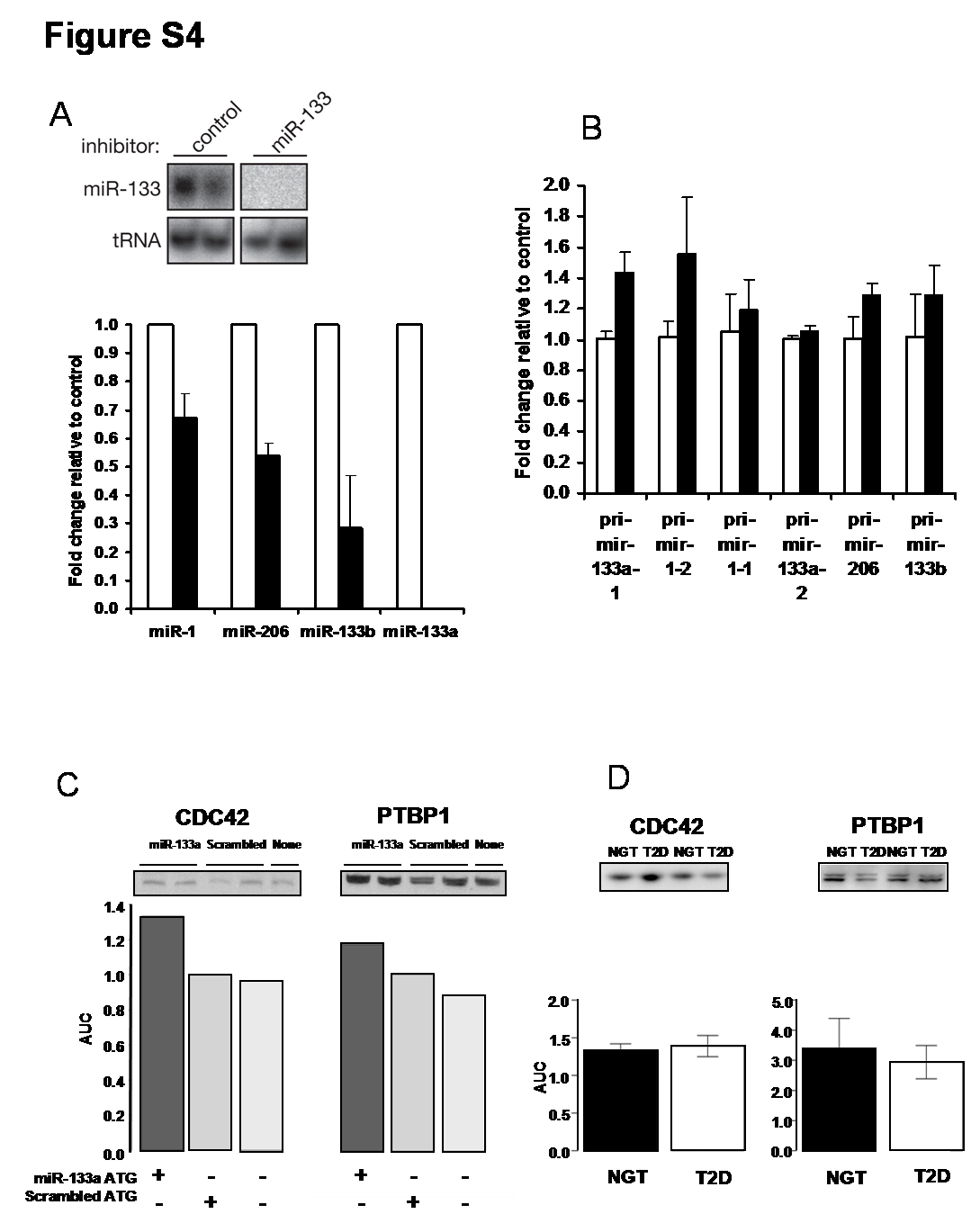


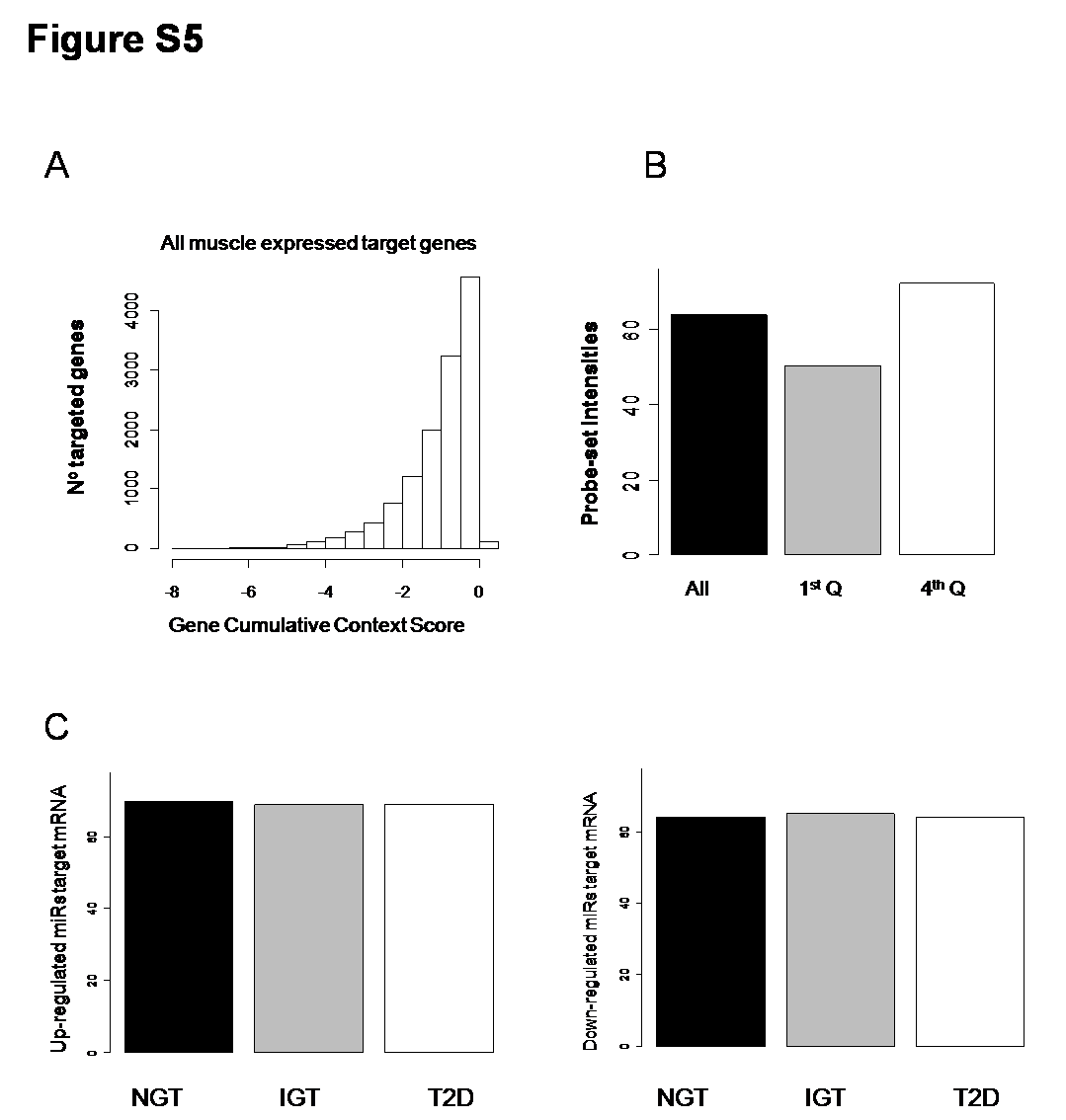


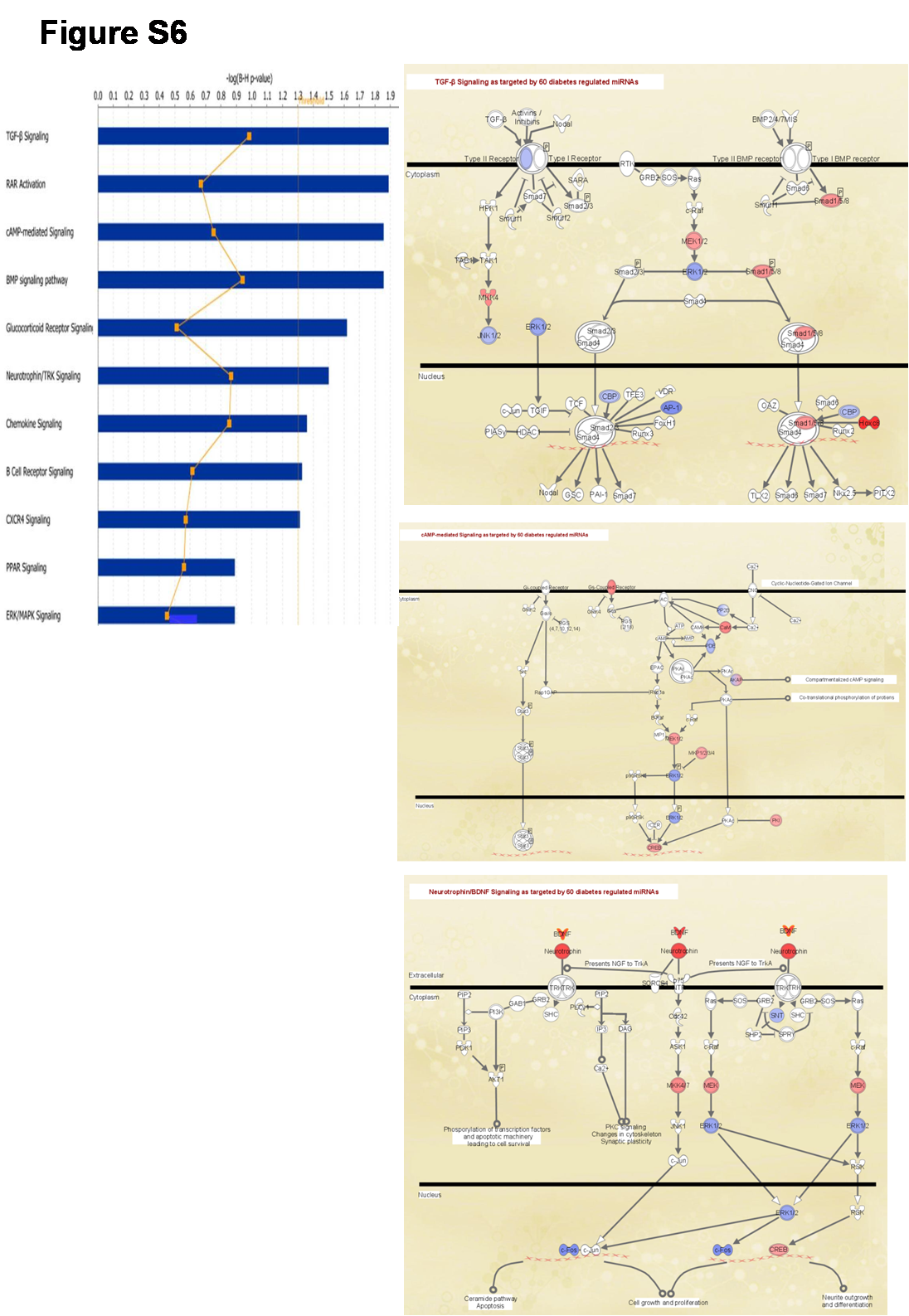

Supplement: Additional file 1 — Figures S1 to S6, Tables S1 to S4 and supplementary Results and Discussion. [file gm130-S1.doc]
